# NFIC mediates m6A mRNA methylation to orchestrate transcriptional and post-transcriptional regulation to represses malignant phenotype of non-small cell lung cancer cells

**DOI:** 10.1186/s12935-024-03414-1

**Published:** 2024-06-28

**Authors:** Kesong Shi, Yani Chen, Ruihua Liu, Xinyao Fu, Hua Guo, Tian Gao, Shu Wang, Le Dou, Jiemin Wang, Yuan Wu, Jiale Yu, Haiquan Yu

**Affiliations:** State Key Laboratory of Reproductive Regulation and Breeding of Grassland Livestock, School of Life Sciences, Inner Mongolia University, Hohhot, 010020 Inner Mongolia China

**Keywords:** NFIC, M6A modification, METTL3, KAT2A, NSCLC, Transcriptional regulation, Epigenetics

## Abstract

**Background:**

Multiple genetic and epigenetic regulatory mechanisms are crucial in the development and tumorigenesis process. Transcriptional regulation often involves intricate relationships and networks with post-transcriptional regulatory molecules, impacting the spatial and temporal expression of genes. However, the synergistic relationship between transcription factors and N6-methyladenosine (m6A) modification in regulating gene expression, as well as their influence on the mechanisms underlying the occurrence and progression of non-small cell lung cancer (NSCLC), requires further investigation. The present study aimed to investigate the synergistic relationship between transcription factors and m6A modification on NSCLC.

**Methods:**

The transcription factor NFIC and its potential genes was screened by analyzing publicly available datasets (ATAC-seq, DNase-seq, and RNA-seq). The association of NFIC and its potential target genes were validated through ChIP-qPCR and dual-luciferase reporter assays. Additionally, the roles of NFIC and its potential genes in NSCLC were detected in vitro and in vivo through silencing and overexpression assays.

**Results:**

Based on multi-omics data, the transcription factor NFIC was identified as a potential tumor suppressor of NSCLC. NFIC was significantly downregulated in both NSCLC tissues and cells, and when NFIC was overexpressed, the malignant phenotype and total m6A content of NSCLC cells was suppressed, while the PI3K/AKT pathway was inactivated. Additionally, we discovered that NFIC inhibits the expression of METTL3 by directly binding to its promoter region, and METTL3 regulates the expression of KAT2A, a histone acetyltransferase, by methylating the m6A site in the 3’UTR of KAT2A mRNA in NSCLC cells. Intriguingly, NFIC was also found to negatively regulate the expression of KAT2A by directly binding to its promoter region.

**Conclusions:**

Our findings demonstrated that NFIC suppresses the malignant phenotype of NSCLC cells by regulating gene expression at both the transcriptional and post-transcriptional levels. A deeper comprehension of the genetic and epigenetic regulatory mechanisms in tumorigenesis would be beneficial for the development of personalized treatment strategies.

**Supplementary Information:**

The online version contains supplementary material available at 10.1186/s12935-024-03414-1.

## Background

Lung cancer remains the most commonly diagnosed cancer worldwide, with non-small cell lung cancer (NSCLC) accounting for approximately 80% to 90% of all cases [1, 2]. Lung adenocarcinoma (LUAD) and squamous cell carcinoma (LUSC) are the main histological subtypes of NSCLC [2]. Despite advancements in diagnostic and therapeutic approaches, the prognosis for NSCLC patients remains unfavorable [2]. There is still a need to investigate the molecular pathogenic mechanisms and identify new biomarkers to understand and address the challenges of resistance and individual-specific reactions. In recent years, the potential influence of genetic and epigenetic regulatory mechanisms at the levels of transcription and post-transcription on the development of human diseases has garnered growing attention in research [3–5]. Therefore, further studies the synergy between genetic and epigenetic regulatory mechanisms may offer valuable insights for the prevention of NSCLC.

Extensive research has been conducted on the involvement of various epigenetic mechanisms, such as DNA methylation and histone modifications, which significantly contributed to the development and progression of cancer [6]. Moreover, DNA methyltransferase inhibitors (DNMTis) and histone deacetylase inhibitors (HDACis), which have been proven to be greatly beneficial in the treatment of NSCLC [7, 8]. N6-methyladenosine (m6A) is currently a prominent focus of research in the field of epigenetic regulation [4]. It is catalyzed by writers, including METTL3/14/16, WTAP, KIAA1429, ZC3H13, ZC3H4, CBLL1, RBM15/15B, while it is removed by erasers, such as FTO and ALKBH5 [9]. The m6A modification assumes a crucial role in post-transcriptional regulation and exerts a profound influence on progression of cancer [10, 11]. In addition, m6A and other epigenetic modifications have been found to exhibit synergistic effects [12, 13]. Recently, it has been found that RNA m6A plays a regulatory role in influencing transcription by facilitating DNA demethylation and promoting chromatin accessibility [14]. Our group also found that the m6A methyltransferase METTL3 exerts synergistic effects on m6A methylation and histone modification to regulate the function of VGF in lung adenocarcinoma [15]. Notably, emerging evidence indicates that transcription factor-mediated epigenetic control is obligatory for initiating and sustaining transformation and tumorigenesis [16, 17]. Furthermore, the interaction between transcription factors and the epigenetic regulatory machinery regulates the expression of certain gene classes [16]. However, the interplay between transcriptional regulation governed by TFs and post-transcriptional regulation catalyzed by m6A in the progression of NSCLC remains unclear.

In the current study, based on multi-omics data, we screened functional molecules involved in the occurrence and development of NSCLC. Among these molecules, we specifically investigated the role of the transcription factor NFIC, and examined its regulatory network at both the transcriptional and post-transcriptional levels. Our study explored the synergistic effects of TFs and m6A, providing a scientific foundation for identifying new therapeutic targets in NSCLC.

## Materials and methods

### Data sources

The chromatin property data for five samples of human normal lung tissue were obtained from the dataset GSE18927. Additionally, the chromatin property data for 12 samples each of LUAD and LUSC tissue were obtained from the study conducted by Wang et al. [18]. The TCGA-LUAD and TCGA-LUSC dataset was obtained from the GDC Data Portal (https://portal.gdc.cancer.gov/). The NSCLC dataset, which comprises 199 NSCLC tissues and 19 normal lung tissues, was obtained from GSE81089. The MeRIP-seq data of three pairs of lung adenocarcinoma samples and their corresponding tumor-adjacent normal tissues were obtained from the GSE198288 dataset. Moreover, the MeRIP-seq data for METTL3-knockdown in A549 cells were collected from the GSE55572 dataset. The RNA-seq data of NSCLC cell lines (A549 and H460) and human bronchial epithelial cells HBE were derived from GSE200370 and GSE101993 datasets, respectively.

### Bioinformatics analysis

For the chromatin property data (ATAC-seq and DNase-seq), the raw reads were obtained as described above. Detailed procedures for data processing can be found in our previous studies [19]. The peaks were merged into a single file using the bedtools software v2.30.0. Differential peak analysis was conducted using the DiffBind package, applying screening criteria of |fold change|> 3 and false discovery rate (*FDR*) < 0.01. Additionally, the list of transcription factors (TFs) potentially binding to the peaks was identified using the HOMER software [20]. The TFs found were further filtered based on a *p value* < 1 × 10^6^. For MeRIP-seq data, the comprehensive data processing workflow is described in detail in our prior study [15]. For RNA-seq data, differential gene expression analysis was performed with the limma package in the R programming language, applying a *p value* cutoff of < 0.05 and |log2 fold change |> 1. WGCNA was carried out as previously described [19]. The functional annotation and pathway enrichment was conducted using DAVID [21]. Furthermore, Kyoto Encyclopedia of Genes and Genomes (KEGG) pathway and Gene Ontology (GO) analyses were generated using ggplot2 v3.3.0 package.

### NSCLC tissues

The 60 pairs of NSCLC tissues and their corresponding adjacent normal tissues (HLugA060PG02 and HLugSqu090Lym01) were purchased from Shanghai Outdo Biotech Company (Shanghai, China). The cDNA microarray of NSCLC tissues (HColA095Su02) was also purchased from Shanghai Outdo Biotech Company, included 15 samples of NSCLC tissues and their adjacent noncancerous tissues. Ethical approval for this study involving human participants was granted by the Ethics Committee of Shanghai Outdo Biotech Company (approval number: YB M-05-02).

### Cell culture and cell transfection

Two NSCLC cell lines, A549 and H460, as well as a human normal lung epithelial cell line HBE (ATCC, Manassas, USA; Date: January 2023), were cultured at 37 °C in a 5% CO2 incubator. A549 cells were cultured in Dulbecco's Modification of Eagle's Medium (DMEM) supplemented with 10% fetal bovine serum. Meanwhile, H460 and HBE cells were cultured in RPMI 1640 medium (VivaCell, Shanghai, China) also supplemented with 10% fetal bovine serum. Cell identity was confirmed by short tandem repeat (STR) analysis, and Mycoplasma testing was negative (Date: January 2023).

To construct the overexpression vector, the pIRES2-ZsGreen1 vector was employed, and the full-length sequences of NFIC and METTL3 were introduced into pIRES2-ZsGreen1. The METTL3 shRNA, METTL3 siRNA, KAT2A shRNA, KAT2A siRNA, as well as their respective control shRNAs or siRNAs, were provided by Genepharma GenePharma (Shanghai, China). These were individually transduced into A549 and H460 cells using JetPRIME reagent (Polyplus-transfection, France). The efficiency of transfection was confirmed through qRT-PCR and western blot assays. The sequences of the shRNAs and siRNAs used are provided in Additional file 1: Table S1.

### Quantitative real-time polymerase chain reaction (qRT-PCR) and western blot

Total RNA was extracted from cells using the TRIzol reagents (Invitrogen, USA). Subsequently, the RNA was reverse transcribed to cDNA using the PrimeScript RT reagent Kit (RR047A, Takara, Japan). qRT-PCR was performed using the ABI 7500 instrument (Applied Biosystems, USA). The relative transcription level of the target gene was determined using the 2^−ΔΔCt^ method. The primer sequences are provided in Additional file 1: Table S2.

For western blotting analysis, the samples were lysed using RIPA buffer (R0010, Solarbio, China) supplemented with protease inhibitors (Solarbio, China). Equal amounts of proteins were subsequently separated using 10% SDS-PAGE and transferred onto a 0.45 μm PVDF membrane (IPVH00010, Immobilon-P, Millipore). The membrane was then blocked with 5% non-fat milk for 1 h at room temperature. Protein detection was conducted by incubating the membrane with primary antibodies at 4 °C for 16 h, and subsequently incubating it with a secondary antibody for 2 h at room temperature. Finally, protein blotting was visualized using western blot detection reagents (Bio-Rad, CA, USA) and the signal was detected using chemiluminescence with Tanon-5200 (Tanon, Shanghai, China). The primary antibodies used in the study were as follows: phospho-PI3K P85 (TA3242, Abmart, Shanghai, China); PI3K (T40115, Abmart, Shanghai, China); METTL3 (ab195352, Abcam, UK). Additional antibodies including AKT, phospho-AKT, KI67, KAT2A, NFIC, and GAPDH were obtained from Proteintech Biotechnology (Wuhan, China). The original western blot images can be found in Additional file 2.

### Immunohistochemistry

The tissues were de-waxed and sliced. Next, the sections were incubated overnight at 4 °C with primary antibodies specific to NFIC, KAT2A, KI67, or METTL3. After that, biotin-labeled secondary antibodies were applied and left to incubate at 37 °C for 1 h. Finally, representative images were acquired using a microscope from Nikon, Japan.

### Chromatin immunoprecipitation and MeRIP-qPCR assay

Chromatin immunoprecipitation (ChIP) was carried out using the Simple ChIP Enzymatic Chromatin IP Kits (9003S, CST, USA). A549 and H460 cells were treated with formaldehyde for 10 min to facilitate the formation of DNA–protein crosslinks. Subsequently, the cell lysates were sonicated and subjected to immunoprecipitation with an NFIC antibody or IgG as a control. The resulting chromatin DNA was then extracted and analyzed through qRT-PCR. In addition, the Percent Input Method was used to analyze the IP efficiency, and signals obtained from each immunoprecipitation are expressed as a percent of the total input chromatin (Percent Input = 2% × 2^(C[T] 2%Input Sample – C[T] IP Sample)^). MeRIP-qPCR assays were conducted following our previously published protocols [15]. The primers used in both ChIP-qPCR and MeRIP-qPCR can be found in Additional file 1: Table S3.

### Luciferase reporter assay

Wild-type and mutant KAT2A-3′UTR fragments containing m6A motifs were synthesized by Beyotime (Shanghai, China) and inserted into pmirGLO (Promega, Wisconsin, USA) reporter vectors. Cells were seeded into 6-well plates at a confluence of 70%. Following overnight incubation, cells were co-transfected with vectors. Following a 48-h transfection period, luciferase activity was assessed using the Dual-Luciferase Reporter Assay System (Promega, Wisconsin, USA).

### RNA m6A quantification

Total RNAs were isolated from NSCLC cells using TRIzol (Invitrogen, USA) according to the manufacturer's instructions. The m6A RNA Methylation Assay kit (ab185912, Abcam) was utilized to measure the total m6A content. Briefly, 200 ng of RNA samples were added to the assay wells, Subsequently, the capture antibody, detection antibody, and enhancer solution were introduced in a sequential manner. The m6A levels were measured by reading the absorbance at 450 nm.

### CCK-8 assay and Edu assay

Cell proliferation was assessed using the CCK-8 method (FC101, TransGen, Beijing, China). The transfected A549 and H460 cells were seeded in 96-well plates at a density of 1 × 10^4^ cells per well and counted every 24 h for 3 days. For the EdU incorporation assay (EdU), cells were seeded in 6-well plates at a density of 2 × 10^5^ cells per well and cultured with the EdU reagent (C0075s, Beyotime, China) for 2 h the following day. Subsequently, the cells were fixed with 4% paraformaldehyde and stained with a fluorescent dye. The ImageJ software was utilized to count the number of EdU-positive cells.

### Colony formation assays

Colony formation assays were conducted using 6-well plates, with 500 cells per well. The transfected cells were cultured in medium for approximately 14 days, with a change of medium every 2 days. After that, the colonies were fixed with 4% formaldehyde for 10 min and stained with crystal violet for 15 min. The number of visible colonies was manually calculated.

### Wound healing assay

In order to evaluate the cell migration properties of A549 and H460 cells, which were plated in 6-well plates (1 × 10^6^ cells per well), a scratch was created on the cell layer using a 200 μl pipette tip to form a wound. Images of the wounds were captured using a light microscope (Olympus, Tokyo, Japan) at 0 and 24 h after the wounds were made. The closure of the wounds was subsequently assessed using ImageJ software. The in vitro wound-healing potential was evaluated by calculating the percentage of the wound-healing rate (distance migrated / original wound distance × 100%).

### Transwell assay

Transwell chambers (Corning, New York, USA) were utilized to assess the invasive or migratory capability of NSCLC cells. Briefly, A549 and H460 cells were seeded in the upper chambers of transwell plates coated with or without Matrigel (356234, Corning, Acton, Massachusetts, USA).

### In vivo* tumor formation assay*

Female BALB/c nude mice (4 weeks old) were obtained from Beijing SPF (Beijing, China) and were randomly assigned to groups, with each group consisting of 6 mice. To ensure the random allocation of mice into the control and experimental groups, all experimental mice were initially assigned numbers and randomized using a random number table. Consistency was also maintained across factors such as age, environmental conditions, and feeding conditions of the mice. Furthermore, researchers were unaware of the specific group assignment of each mouse, mitigating potential subjective biases. The transfected A549 cells (1 × 10^7^) were then subcutaneously implanted into the flank of each mouse. One month after transplantation, the mice were humanely sacrificed and their tumors were excised for both weighing and histological analysis. The tumor volume was calculated using the formula 1/2 × length × width^2^. All the mice were housed at the Animal Center of Inner Mongolia University, China, and the study protocol was approved by the Animal Care and Use Committee of Inner Mongolia University (approval ID: IMU-mouse-2022-053).

### Statistical analysis

Statistical analyses were conducted using GraphPad Prism 8.0.2 software (GraphPad Software, United States), and the results are presented as the mean ± standard deviation (SD). Each experiment was performed three times, unless otherwise specified. Significant differences were assessed by two‐tailed Student's t‐test or one-way ANOVA for comparisons between multiple groups.

## Results

### Screening of transcription factors associated with NSCLC

**Fig. 1:**
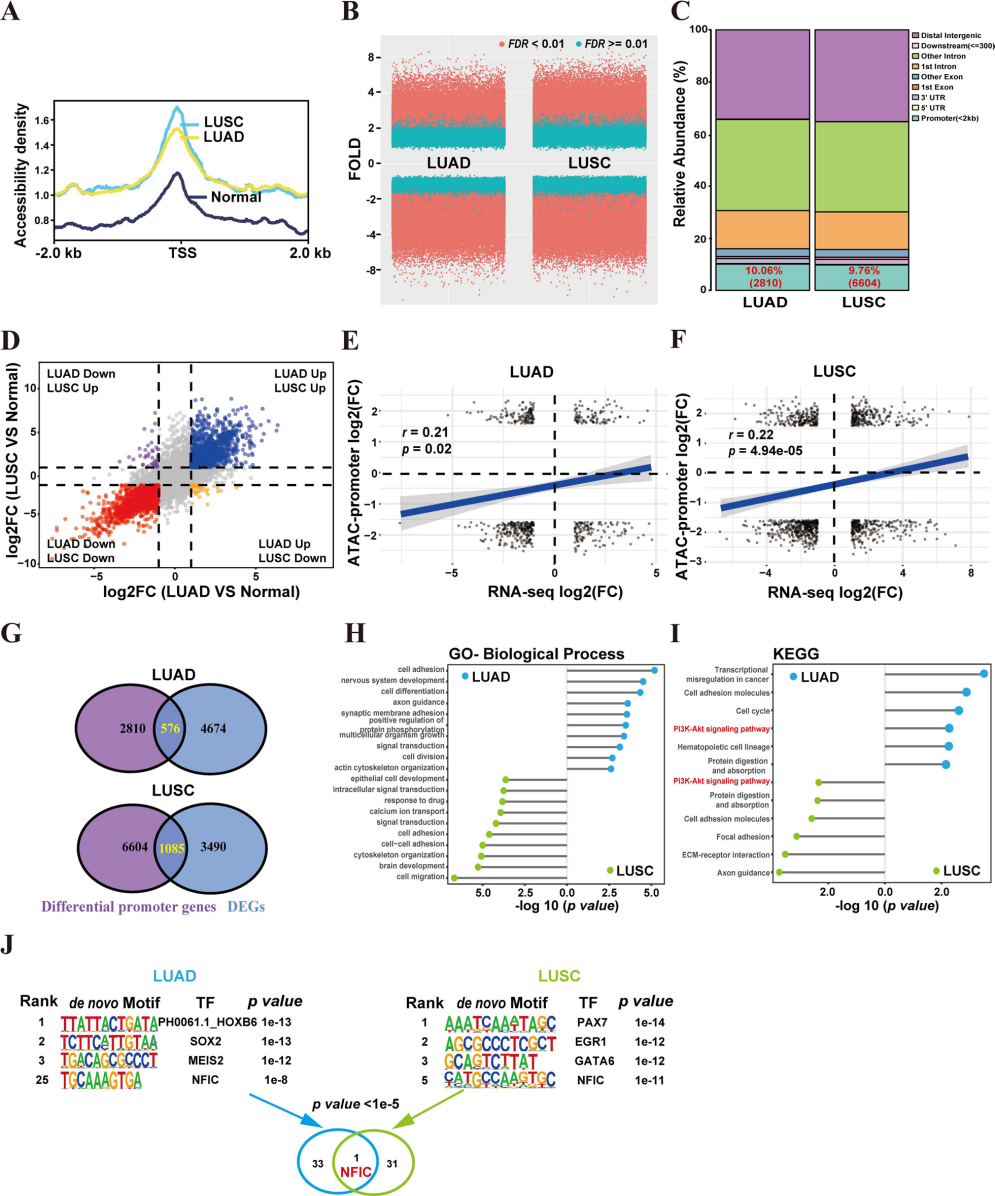
Screening of transcription factors associated with NSCLC. **A** Line plot shows the chromatin accessibility in NSCLC and normal tissue around the TSS of the nearest genes. **B** Volcano plot showing differential expressed peaks between NSCLC and normal tissue. **C** Relative distribution of gene coding regions, introns, exons, and upstream and downstream regions. **D** Differential gene expression analysis of NSCLC. **E–F** Point density plots indicating the correlation between promoter chromatin accessible regions and gene expression in LUAD (**E**) and LUSC (**F**). **G** Venn diagram showing the overlapping number of differential genes. **H-I** GO biological process (**H**) and KEGG pathway (**I**) analysis of overlapping differential genes. **J** Overlapping TF motif of LUAD and LUSC

The significance of open chromatin-accessible regions containing crucial genomic elements for transcription factor (TF) binding and gene regulation has been acknowledged [22, 23]. The assay for transposase-accessible chromatin followed by sequencing (ATAC-seq) and Deoxyribonuclease I (DNase I)-hypersensitive site sequencing (DNase-seq) has been widely used to measuring open regions of chromatin [24, 25]. To search for TFs associated with NSCLC, we initially conducted an analysis of chromatin accessibility profiling in human normal lung tissues, as well as LUAD and LUSC tissues, utilizing publicly available data (ATAC-seq, DNase-seq). A substantial number of accessible peaks were found near the transcription start site (TSS) in LUAD, LUSC, and normal lung tissues (Fig. 1A), indicating a propensity for binding to transcription factors. Additionally, compared to normal lung tissues, we found 33,215 and 45,630 differentially accessible peaks in LUAD and LUSC tissues, respectively (fold change >|3.5|, false discovery rate FDR < 0.01) (Fig. 1B). Among these, 10.06% and 9.76% of the differential accessible peaks were located in the promoter region (2 kb region upstream and downstream), respectively (Fig. 1C). Subsequently, these differential accessible peaks of promoter region were annotated to the nearest gene. After removing duplicate genes, we obtained 2810 and 6604 genes in LAUD and LUSC, respectively (Fig. 1C).

Given the complexity of gene expression regulation, it is necessary to explore biological questions from different perspectives. Therefore, multi-omics analysis is becoming increasingly important. We examined the differentially expressed genes (DEGs) in LUAD, LUSC, and para-tumor samples from the TCGA RNA-seq database. Comparative analysis revealed 4,674 DEGs in LUAD and 3,490 DEGs in LUSC when compared to the para-tumor samples (Fig. 1D). Subsequently, we assessed the correlation between gene expression and chromatin accessibility. Significantly positive correlations were observed between gene expression and promoter accessibility in LUAD (correlation coefficient *r* = 0.21, *p value* = 0.02) and LUSC (*r* = 0.22, *p value* = 4.94e-05) (Fig. 1E–F). By overlapping the differentially accessible promoters identified by ATAC-seq and the DEGs from RNA-seq, we obtained a total of 576 and 1,085 overlapping genes in LUAD and LUSC, respectively (Fig. 1G).

To study the role of these overlapping genes in the progression of NSCLC, Gene Ontology (GO) analysis was performed. The analysis revealed enriched categories in fundamental biological processes for the 576 overlapping genes in LUAD, such as cell adhesion, cell differentiation, and synaptic membrane adhesion (Fig. 1H). Likewise, for the 1085 overlapping genes in LUSC, 65 functional terms in the biological process category were found, including cell migration, cytoskeleton organization, and cell adhesion. In addition, KEGG enrichment chart of overlapping genes in LUAD and LUSC were conducted (Fig. 1I). The analysis of LUAD identified 6 enriched functional clusters, including transcriptional mis-regulation in cancer, cell adhesion molecules, cell cycle, PI3K-AKT signaling pathway, hematopoietic cell lineage, and protein digestion and absorption. The analysis of LUSC revealed 6 enriched functional clusters, including axon guidance, ECM-receptor interaction, focal adhesion, cell adhesion molecules, protein digestion and absorption, and PI3K-AKT signaling pathway. These findings provide evidence of the critical roles played by overlapping genes in NSCLC tumorigenesis and metastasis. Furthermore, we performed an analysis of possible transcription factor (TF) motifs in the overlapping genes using the de novo TF motif discovery software HOMER [20]. We identified a total of 33 and 31 TF motif candidates enriched at the promoter regions of overlapping differential genes in LUAD and LUSC, respectively. Notably, only one TF (NFIC) was found to be common between LUAD and LUSC (Fig. 1J). These results suggest that NFIC may play a role in regulating the development and progression of NSCLC.

### NFIC overexpression inhibits the malignant phenotypes of NSCLC cells by inactivating the PI3K/AKT pathway

**Fig. 2:**
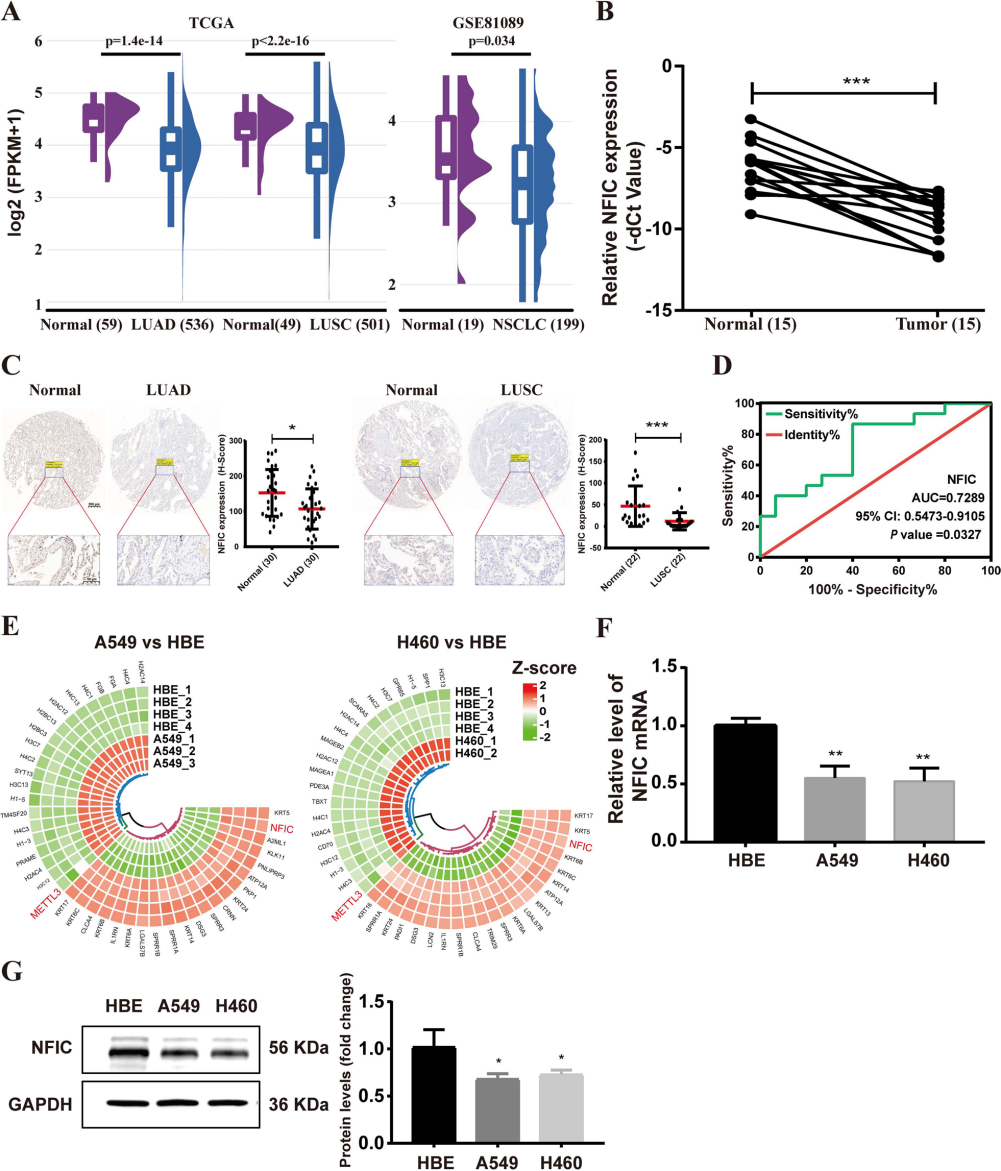
Analysis of NFIC expression. **A** NFIC expression in NSCLC tissues and adjacent normal tissues from the TCGA and GSE81089 datasets. **B**-**C** NFIC expression levels in tumor tissues were detected by qRT-PCR (**B**) and immunohistochemical (**C**). **D** ROC curve analysis of the NFIC gene. **E** Differentially expressed genes in A549 and H460 cells compared to HBE cells. **F-G** NFIC mRNA expression and protein expression patterns in NSCLC cells and HBE cells were measured by qRT-PCR (**F**) and western blot analysis (**G**), respectively. Bar = mean ± SD. **P < 0.01, ***P < 0.001

In order to verify the above hypothesis, we first analyzed the expression pattern of NFIC in LUAD and LUSC using TCGA and GEO (GSE81089) data. The results demonstrated a significant downregulation of NFIC in tumor tissues compared to normal tissues (Fig. 2A). This downregulation was further confirmed through qRT-PCR (Fig. 2B) and immunohistochemistry (IHC) analysis (Fig. 2C). Additionally, we utilized a receiver operating characteristic (ROC) curve to assess the diagnostic potential of NFIC as a biomarker for NSCLC. Figure 2D showed that NFIC has an area under the ROC curve (AUC) of 0.7289, suggesting its ability to distinguish between NSCLC and normal tissue with good diagnostic efficiency. Meanwhile, we analyzed the RNA-seq data of NSCLC cell lines (A549, H460) and human normal lung epithelial cell lines (HBE) available in the GEO database. The results revealed a significant downregulation of NFIC in A549 and H460 cells compared to HBE cells (Fig. 2E). Consistent with the GEO database, qRT-PCR and western blot analysis further confirmed the decline of NFIC in A549 and H460 cells compared to HBE cells (Fig. 2F–G).

**Fig. 3:**
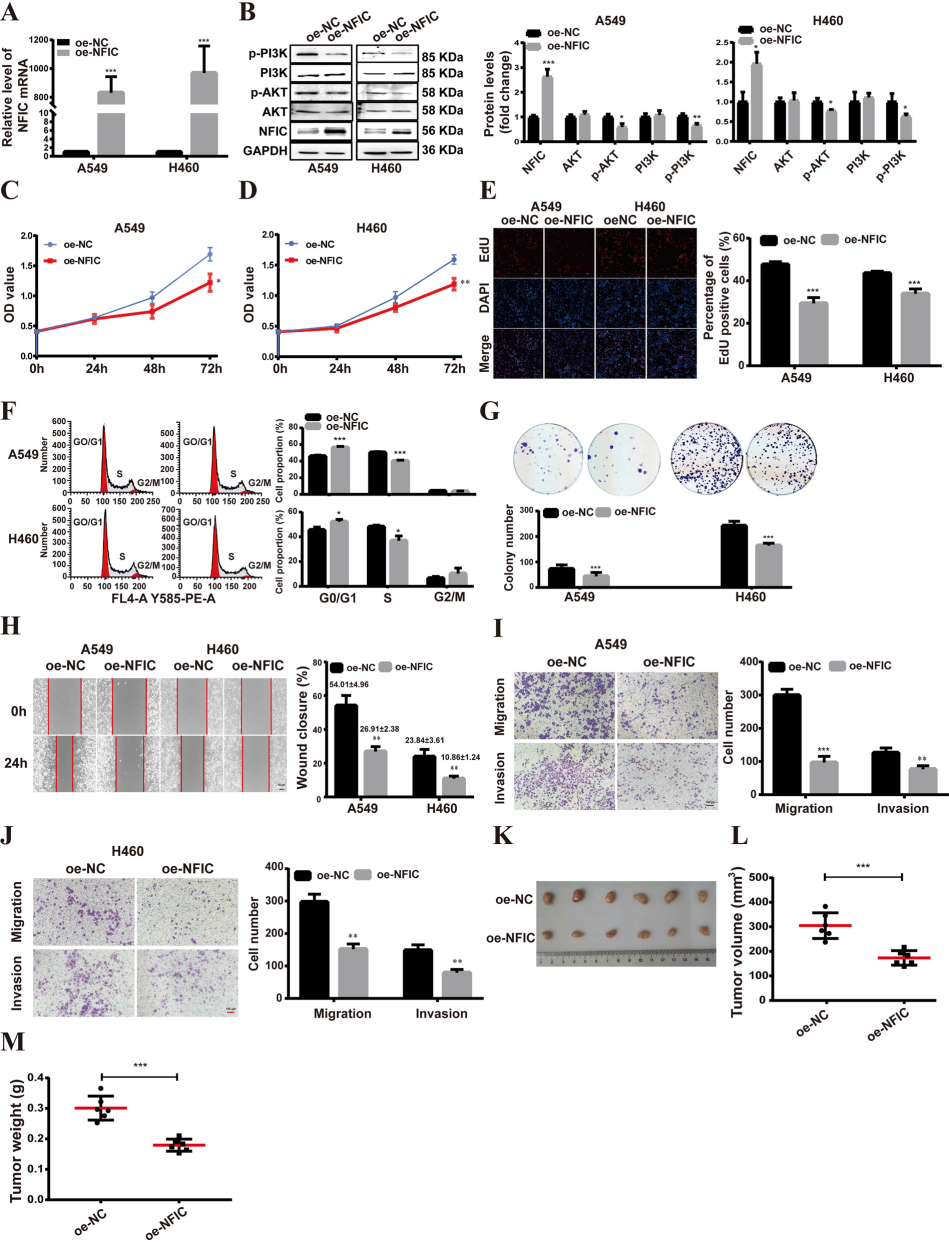
NFIC overexpression inhibits proliferation, migration, and invasion of NSCLC cells. **A** qRT-PCR analysis of NFIC overexpression efficiency in A549 and H460 cells. **B** Western blot analysis for NFIC, PI3K, p-PI3K, AKT, and p-AKT protein expression. **C-D** Proliferation of A549 (**C**) and H460 (**D**) cells following NFIC overexpression was determined using CCK8 assays. **E** EdU assays in A549 and H460 cell lines; scale bars = 100 µm. **F** Analyses of A549 and H460 cell cycle distributions by flow cytometry. **G**. Colony formation assays. **H** Wound-healing assays in A549 and H460 cell lines; scale bars = 100 µm. **I-J** Transwell migration and matrigel invasion assays for A549 (**I**) and H460 (**J**) cells. **K** The effect of NFIC overexpression on NSCLC subcutaneous xenografts in vivo. **L-M** The tumor volume (**L**) and weight (**M**) of tumors xenografted in nude mice. Bar = mean ± SD. *P < *0.05*, **P < *0.01*, ***P < *0.001*

To further explore the function of NFIC in NSCLC, we transfected NFIC overexpression (oe-NFIC) vector into NSCLC (A549, H460) cells. In the qRT-PCR assay, the expression of NFIC was found to be significantly upregulated in A549 and H460 cells (Fig. 3A). The overexpression efficiency of NFIC was verified by western blot assay (Fig. 3B). Moreover, the overexpression of NFIC significantly suppressed the proliferation of A549 and H460 cells as determined via Cell Counting Kit-8 (CCK8) (Fig. 3C, D) and EdU staining (Fig. 3E). We used flow cytometry to analyze cell cycle progression, the data showed that NFIC overexpression caused a dramatic decrease in S-phase and accumulation in G1 phase of A549 and H460 cells (Fig. 3F), and NFIC overexpression markedly reduced colony formation in both A549 and H460 cells (Fig. 3G). Furthermore, the results of the wound healing assays showed that NFIC overexpression led to decreased cell migration (Fig. 3H). Transwell assays showed that the number of migrated and invaded cells decreased in NFIC overexpressing A549 (Fig. 3I) and H460 (Fig. 3J) cells compared to control cells. Subsequently, in vivo experimental results showed that compared to control group, the NFIC overexpression groups displayed smaller tumors and slower tumor growth (Fig. 3K–M).

Previously, overlapping differential genes between chromatin property and RNA-seq data enriched in PI3K/AKT signaling pathway (Fig. 1I). To determine whether the overexpression of NFIC regulates the PI3K/AKT signaling pathway, the effects of NFIC overexpression on PI3K/AKT signaling pathway were investigated by western blot. The results showed that the overexpression of NFIC decreased the phosphorylation level of PI3K and AKT (Fig. 3B). These results suggested that the overexpression of NFIC suppressed the malignant phenotypes of NSCLC cells by inactivating the PI3K/AKT pathway.

### NFIC negatively regulates METTL3 expression in A549 and H460 cells

**Fig. 4:**
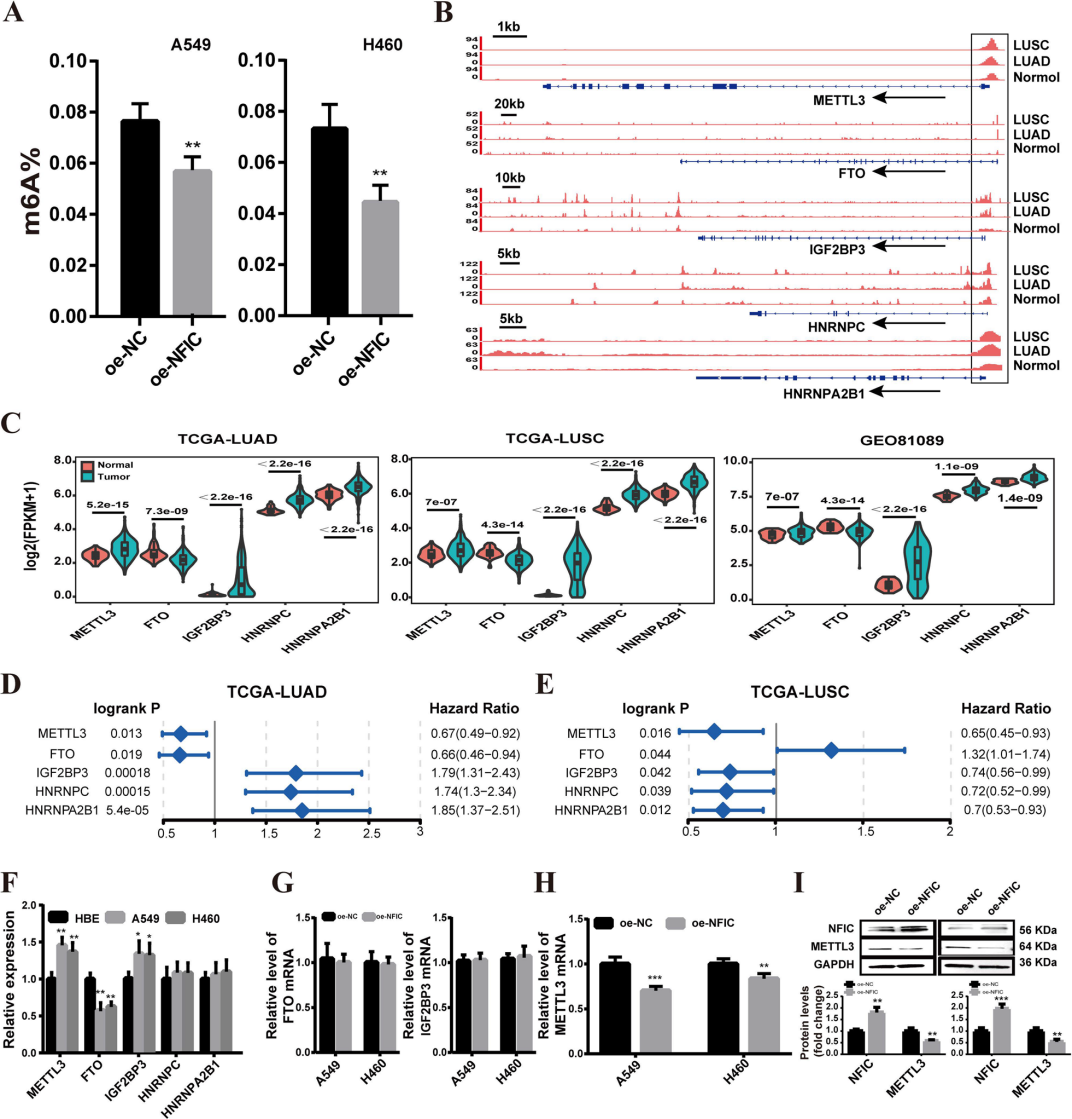
NFIC negatively regulates METTL3 expression in A549 and H460 cells.** A** The total m6A level of A549 and H460 cells after METTL3 overexpression. **B** Integrative Genomics Viewer tracks displaying chromatin accessibility read distributions in m6A-related genes.** C** M6A-related genes expression in NSCLC tissues and normal tissues form the TCGA and GSE81089 datasets. **D-E** Forest map of m6A-related genes on survival analysis in LUAD (**D**) and LUSC (**E**). **F** *METTL3*, *FTO*, *IGF2BP3*, *HNRNPC*, and *HNRNPA2B1* mRNA expression in A549 and H460 cells. **G** The expression of *FTO* and *IGF2BP3* mRNA with NFIC overexpression in A549 and H460 cells. **H-I** qRT-PCR (**H**) and western blot (**I**) analysis of the expression of METTL3 with NFIC overexpression in A549 and H460 cells. Bar = mean ± SD. *P < 0.05, **P < 0.01, ***P < 0.001

Transcription factors, as key regulators of gene transcription, often affect the occurrence and development of cancer through regulating the transcription process of target genes. Therefore, it is necessary to further explore the target genes of the transcription factor NFIC. Recently, M6A modification has emerged as one of the most popular fields in cancer research [26], and a large number of studies have shown that m6A-related genes have been associated with NSCLC [10, 11, 27]. We found that NFIC overexpression resulted in a downregulation of global m6A modification level in both A549 and H460 cells compared to the control (Fig. 4A). Additionally, in the above-mentioned chromatin accessibility data (ATAC-seq, DNase-seq) and RNA-seq data, the promoter region chromatin accessibility of m6A-related genes (*METTL3*, *FTO*, *IGF2BP3*, *HNRNPC*, *HNRNPA2B1*) is increased in NSCLC (Fig. 4B), and these genes exhibited significant differential expression levels in NSCLC tissues compared to normal lung tissues (Fig. 4C). Moreover, as depicted in Fig. 4D, E, *METTL3*, *FTO*, *IGF2BP3*, *HNRNPC*, and *HNRNPA2B1* demonstrated significant associations with prognosis in LUAD and LUSC patients (logrank *p values* < 0.05). Subsequently, we investigated the expression levels of these m6A-related genes in NSCLC (A549, H460) and HBE cells. The results indicated significant expression differences for *METTL3*, *FTO,* and *IGF2BP3* between the A549 and H460 cells relative to HBE cells (Fig. 4F). Further analysis demonstrated that *FTO* and *IGF2BP3* mRNA did not exhibit a significant change with NFIC overexpression compared to the control group in A549 and H460 cells (Fig. 4G). However, overexpression of NFIC significantly decreased METTL3 expression (Fig. 4H, I), suggesting that NFIC negatively regulates the expression of METTL3 in NSCLC cells.

### NFIC overexpression delayed the progression of NSCLC by downregulating METTL3 expression

**Fig. 5:**
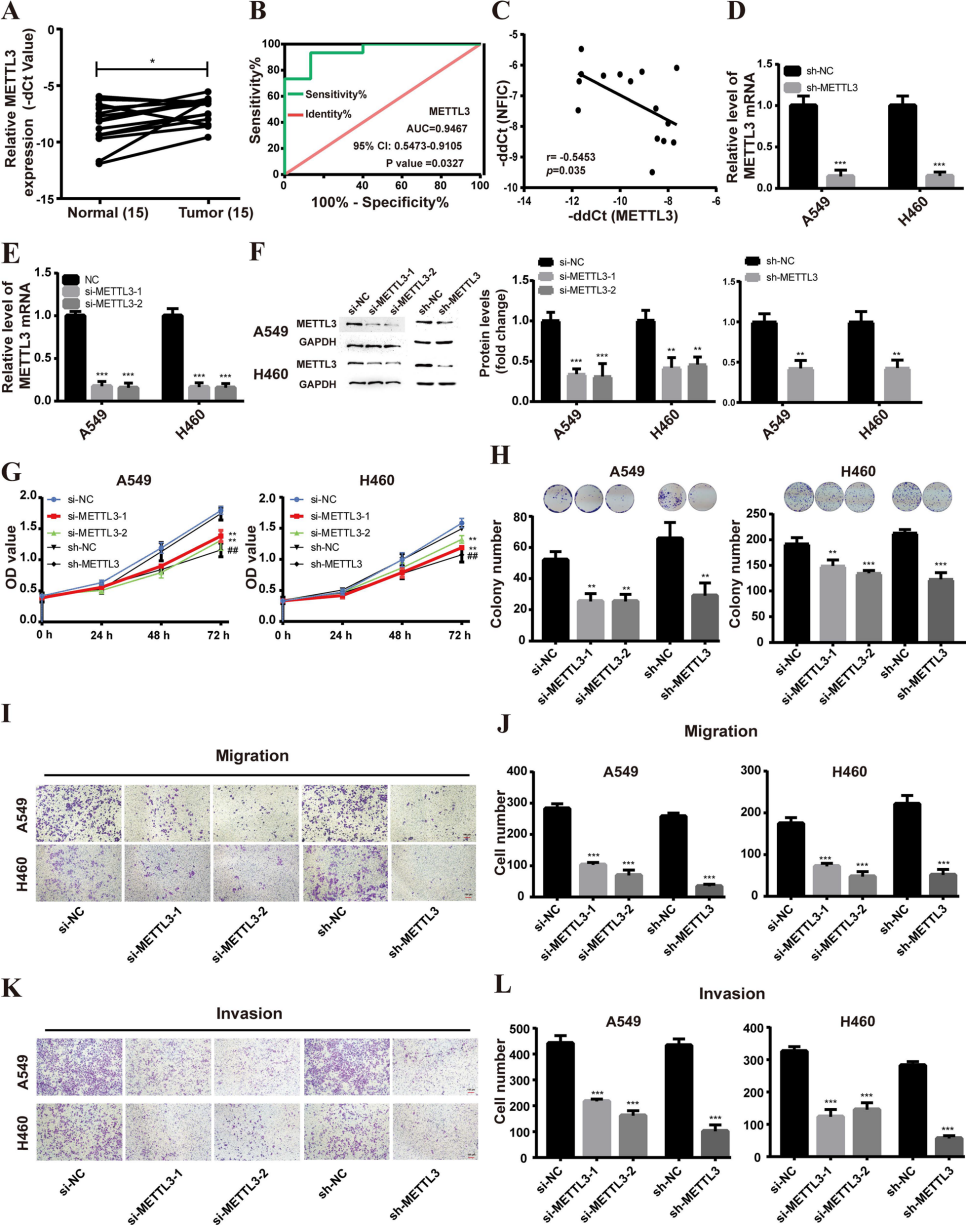
Knockdown of METTL3 inhibited the proliferation, migration, and invasion of NSCLC cells. **A** METTL3 expression levels in tumor tissues were detected by qRT-PCR. **B** ROC curve analysis of the METTL3 gene. **C.** METTL3 and NFIC correlation in NSCLC tissues. **D**-**F** qRT-PCR (**D**-**E**) and western blot (**F**) analysis of METTL3 knockdown efficiency in A549 and H460 cells. **G** Proliferation of A549 and H460 cells following METTL3 knockdown was determined using CCK8 assays. **H** Colony formation assay was performed in A549 and H460 cells after knockdown of METTL3. **I**-**L** Transwell migration assays (**I**-**J**) and invasion assays (**K**-**L**). Bar = mean ± SD. *P < 0.05, **P < 0.01, ***P < 0.001, compared to si-NC group; ^##^*P* < 0.01, compared to sh-NC group

Based on the findings obtained, it was observed that METTL3 exhibited a significantly high expression and a strong correlation with NFIC in NSCLC. To further substantiate these results, the expression of METTL3 was assessed using qRT-PCR. As shown in Fig. 5A, METTL3 was upregulated in NSCLC tissues compared with the adjacent noncancerous tissues (Normal). Moreover, the diagnostic potential of METTL3 as a biomarker for NSCLC was evaluated using a ROC curve. The area under the AUC was determined to be 0.9467 (Fig. 5B), indicating its ability to effectively discern between NSCLC and normal tissue with good diagnostic efficiency. Additionally, an investigation into the gene expression correlation between METTL3 and NFIC was carried out, revealing a negative association between the expression of NFIC and METTL3 in NSCLC tissues (*r* =−0.5453, *p value* = 0.035) (Fig. 5C).

To investigate the impact of METTL3 on the biological functions of NSCLC cells, a transfection experiment was conducted using siRNA-METTL3, shRNA-METTL3, siRNA-control, and shRNA-control in A549 and H460 cells. After 24 h of transfection, qRT-PCR analysis (Fig. 5D, E) and western blot analysis (Fig. 5F) were performed to assess the expression pattern of METTL3 in A549 and H460 cells. Remarkably, both siRNA-METTL3 and shRNA-METTL3 transfection resulted in a significant reduction in METTL3 expression when compared to the control groups. The proliferation of A549 and H460 cells was found to be significantly inhibited upon suppression of METTL3, as demonstrated by the CCK-8 assay (Fig. 5G). Consistently, colony formation assays revealed a notable decrease in the number of cell colonies in A549 and H460 cells upon knockdown of METTL3 (Fig. 5H). Additionally, as depicted in Fig. 5I–L, silencing METTL3 expression led to a substantial decrease in the migratory and invasive abilities of A549 and H460 cells. These findings suggest that knockdown of METTL3 effectively restrains the progression of NSCLC.

**Fig. 6:**
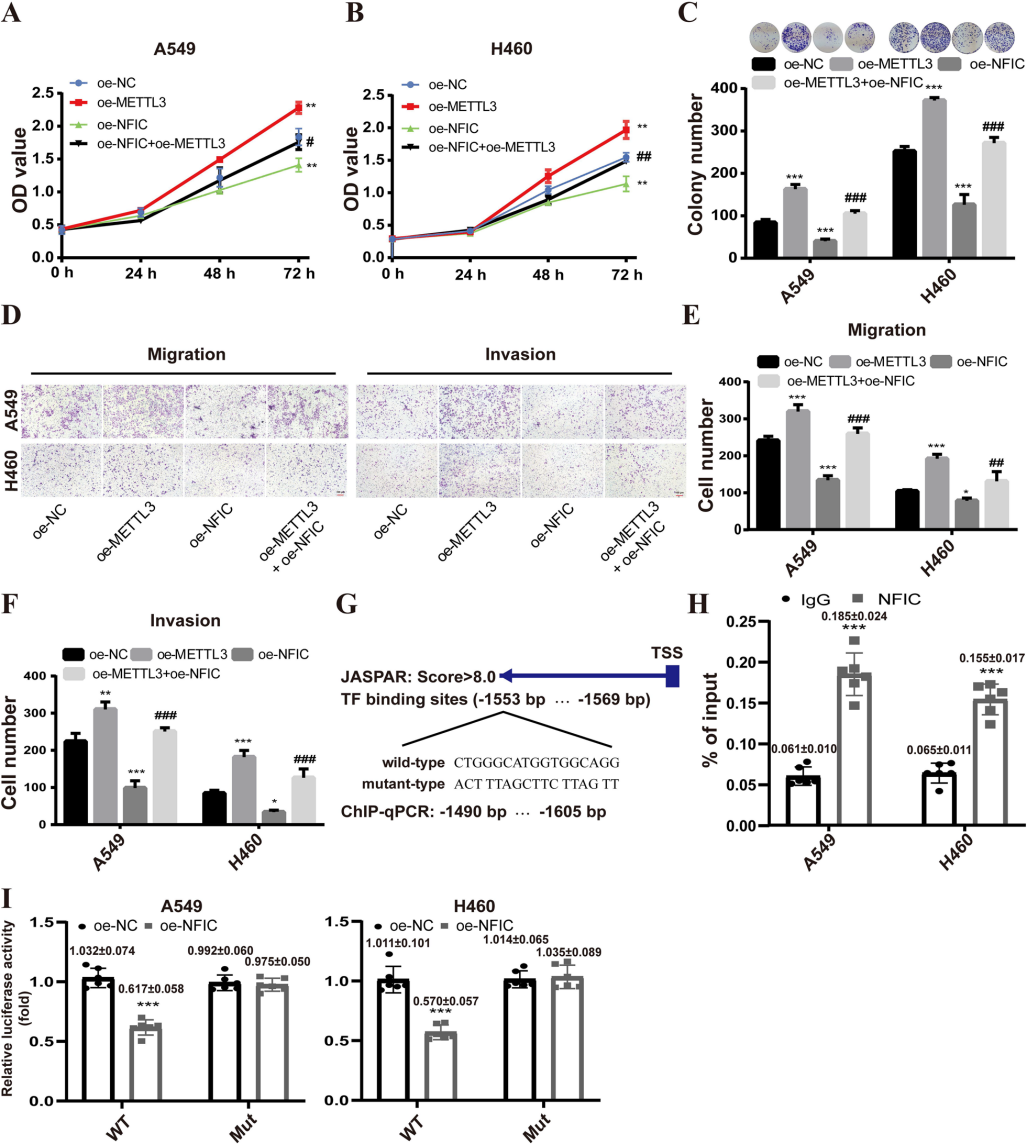
NFIC delayed NSCLC progression via the regulation of METTL3. **A-B** CCK8 assays for A549 (**A**) and H460 (**B**) cells transfected with overexpression plasmid METTL3 alone or overexpression plasmids of both METTL3 and NFIC (METTL3 + NFIC). **C** Colony formation assays for A549 and H460 cells. **D-E** Transwell migration (**D**) and invasion (**E**) assays for overexpression plasmid METTL3 alone or overexpression plasmids METTL3 + NFIC transfected A549 and H460 cells. **F** Prediction results of the binding of NFIC at the site of upstream the TSS of METTL3. **G** ChIP-qPCR detected NFIC binding to METTL3 promoter region in A549 and H460 cells. **H-I** Assessment of METTL3 promoter activity after NFIC overexpression in A549 (**H**) and H460 (**I**) cells via dual‐luciferase reporter assay. *P < 0.05, **P < 0.01, ***P < 0.001, compared to oe-NC group; ^#^*P* < 0.05, ^##^*P* < 0.01, ^###^*P* < 0.001, compared to overexpression METTL3 (oe-METTL3) group

Next, we further investigated whether NFIC regulated the progression of NSCLC by downregulating METTL3 expression. The plasmid for overexpressing METTL3 was transfected into A549 and H460 cells with NFIC overexpression. The results revealed that overexpression of METTL3 reversed the inhibitory effect of NFIC overexpression on NSCLC cell proliferation, colony formation, migration, and invasion (Fig. 6A–E). Furthermore, we used the JASPAR database to predict the binding sites of NFIC on the METTL3 promoter. The analysis indicated a potential binding site of NFIC at the 1553–1569 region upstream of the METTL3 TSS (Fig. 6F). Subsequently, ChIP -qPCR and dual-luciferase reporter assay were performed to further verify the results. The ChIP-qPCR results showed a relative enrichment of NFIC at the METTL3 promoter (Fig. 6G), and dual-luciferase reporter assays showed that overexpression of NFIC decreased the activity of luciferase with wild-type METTL3 but not mutated METTL3 in A549 (Fig. 6H) and H460 (Fig. 6I) cells. These results suggested that the NFIC directly regulated METTL3 expression by binding to the promoter region of METTL3, thereby inhibiting the malignant phenotype of NSCLC cells.

### *METTL3 positively regulates KAT2A mRNA *via* m6A modification*

**Fig. 7:**
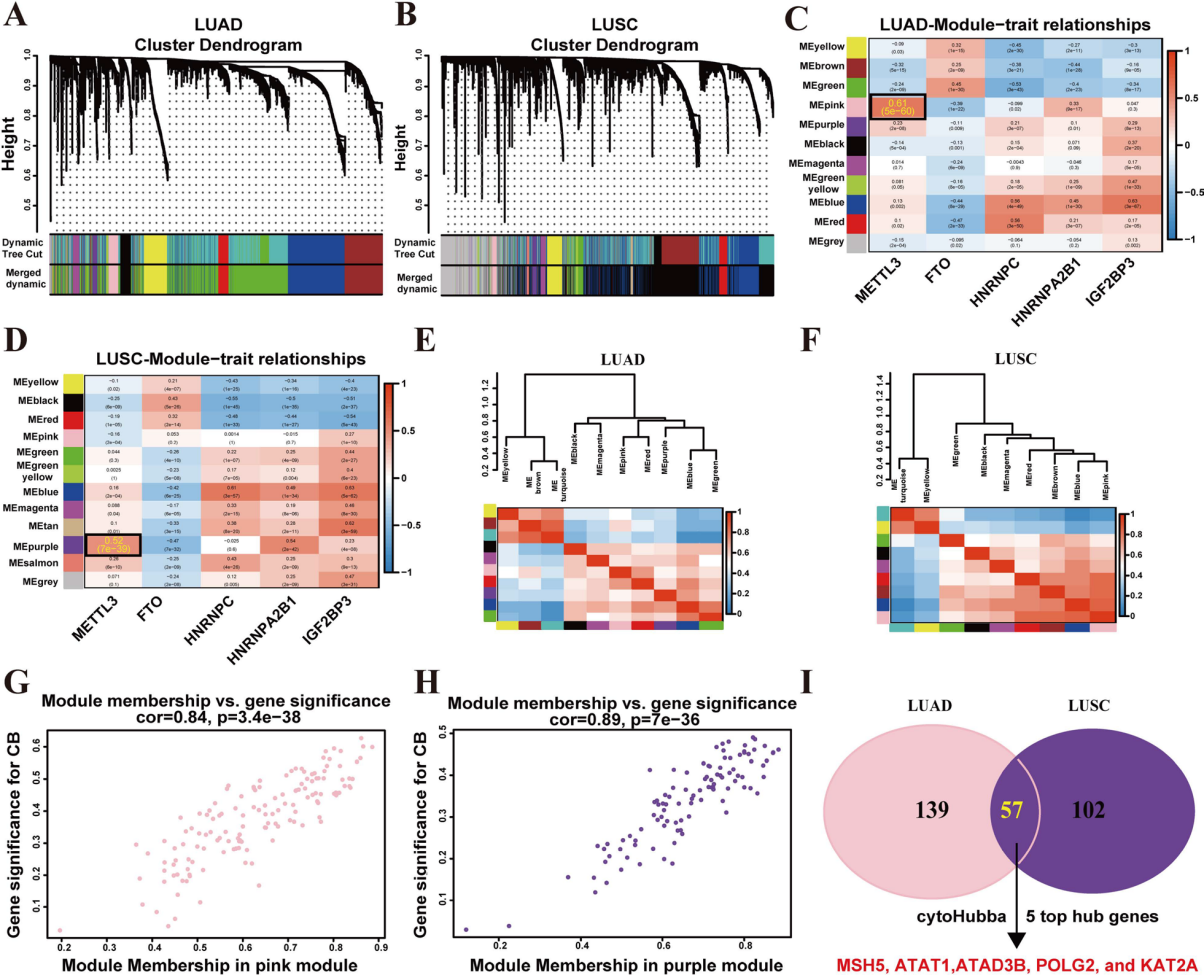
WGCNA of the m6A-related genes. **A-B** Hierarchical clustering tree in LUAD (**A**) and LUSC (**B**). **C-D** The correlation between the gene module and prognosis-related m6A regulators in LUAD (**C**) and LUSC (**D**). **E–F** Clustering module hub genes by hierarchical structure and heatmap of the adjacencies in the hub gene network in LUAD (**E**) and LUSC (**F**). **G-H** Scatter plot of the GS for the grade vs. the MM in the pink (**G**, LUAD) and purple (**H**, LUSC) module. **I** The hub genes of protein–protein interaction (PPI) network of overlapping genes

METTL3 have been studied to regulate cancer progression by regulating target genes through m6A modification [28]. Therefore, we conducted further analysis to identify the target genes that can be regulated by METTL3 through m6A modification in NSCLC. First, weighted correlation network analysis (WGCNA) analysis was performed using prognosis-related m6A regulators and differentially expressed genes of TCGA-LUAD and TCGA-LUSC. The co-expression modules, generated from the scale-free network, were visualized using dynamic tree cutting (Fig. 7A, B). Subsequently, the 11 and 12 modules marked were identified in LUAD and LUSC, respectively. Among them, the pink and purple module were significantly positively (*r* > 0.5, *p value* < 0.01) correlated with the METTL3 in LUAD and LUSC, respectively (Fig. 7C, D). Additionally, we calculated the Pearson’s correlation coefficients between each module and found that each module demonstrated independent validation (Fig. 7E, F). The correlation between the gene significance (GS) and module membership (MM) in the pink (LUAD) and purple (LUSC) module were evaluated. The correlation was significant in the pink (*r* = 0.84, *p value* = 3.4e-38) and purple (*r* = 0.89, *p value* = 7e-36) module (Fig. 7G, H). We also identified 57 overlapping genes in the pink module (LUAD) and purple module (LUSC), and further identified 5 hub overlapping genes using cytoHubba from Cytoscape (https://cytoscape.org/) (Fig. 7I).

**Fig. 8:**
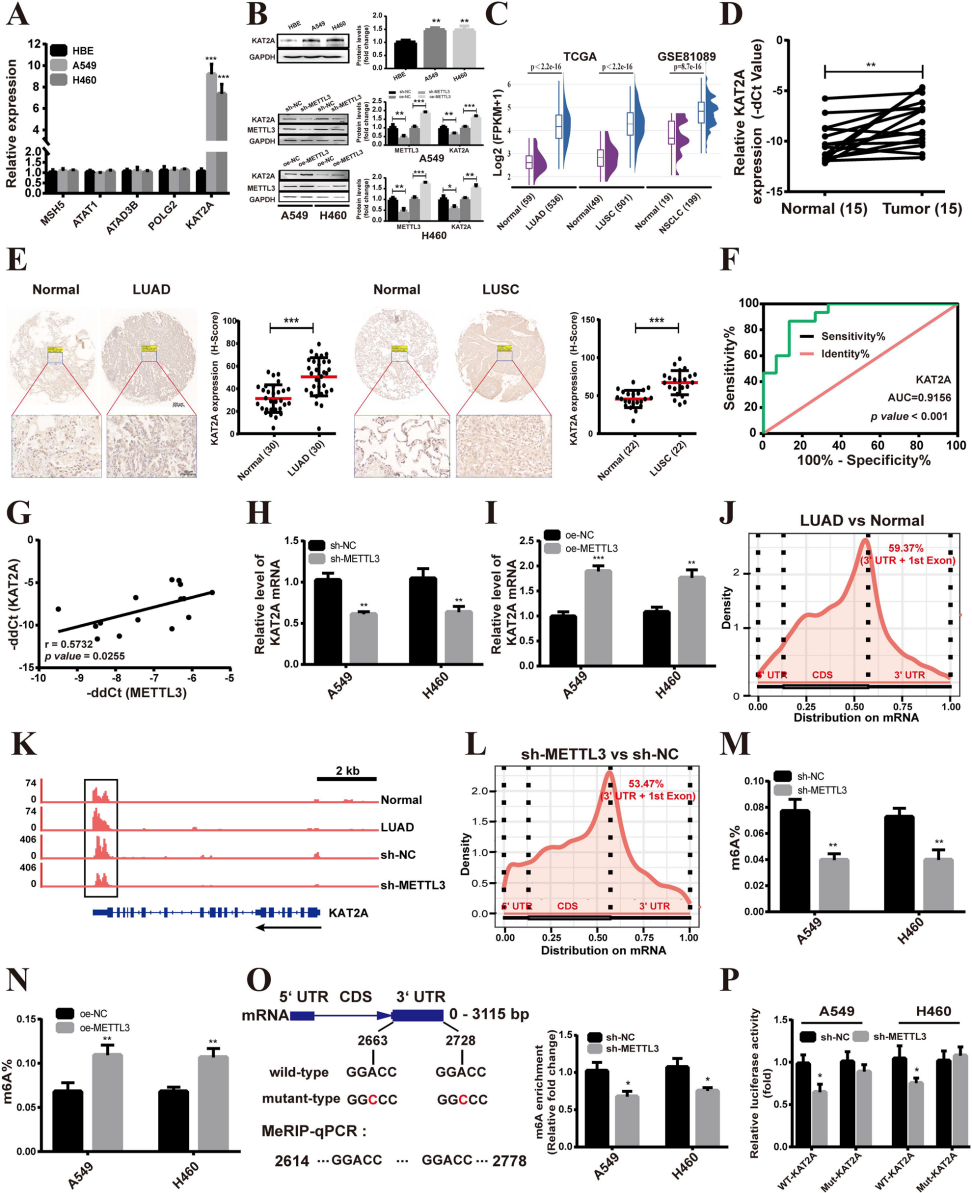
METTL3 positively regulates KAT2A mRNA via m6A modification. **A** qRT-PCR detection of *MSH5*, *ATAT1*, *ATAD3B*, *POLG2*, and *KAT2A* mRNA expression. **B** Western blot results. **C** The relative expression of KAT2A in NSCLC from the TCGA and GSE81089 dataset. **D** qRT-PCR validation of KAT2A expression levels on cDNA microarrays. **E** Representative immunohistochemical staining for KAT2A (scale bar: 200 or 50 μm). **F** ROC curve analysis of the *KAT2A* gene. **G** Correlations between the expressions of METTL3 and KAT2A in NSCLC tissues. **H-I** qRT-PCR analysis of the expression of KAT2A with METTL3 knockdown (**H**) and overexpression (**I**) in A549 and H460 cells. **J** The mRNA density coverage of differential m6A peaks. **K** Display of MeRIP-seq read distributions in KAT2A using Integrative Genomics Viewer. **L** The mRNA density coverage of differential m6A peaks between METTL3-depleted A549 cells and control cells. **M–N** The total m6A level of A549 and H460 cells after METTL3 knockdown (**M**) and overexpression (**N**). **O** Schematic photo of 3’UTR-WT, 3’UTR-mutant in *KAT2A* mRNA (left). MeRIP‐qPCR results of KAT2A m6A modification levels in A549 and H460 cells (right). **P** Dual-luciferase assay result. Bar = mean ± SD. *P < 0.05, **P < 0.01, ***P < 0.001

Next, the expression of hub overlapping genes was examined via qRT-PCR in NSCLC cells (A549, H460) and HBE cells. The results showed that among the tested genes, only KAT2A mRNA expression displayed significant differences in A549 and H460 cells (Fig. 8A). Furthermore, western blot analysis revealed that KAT2A was significantly upregulated in A549 and H460 cells compared to HBE cells (Fig. 8B). Similarly, the TCGA and GSE81089 datasets demonstrated higher expression of KAT2A in NSCLC tissue samples when compared to adjacent normal tissues (Fig. 8C). These observations were further validated using qRT-PCR (Fig. 8D) and immunohistochemistry (Fig. 8E) in NSCLC tissues. To assess the diagnostic potential of KAT2A as a biomarker for NSCLC, an ROC curve was employed. As shown in Fig. 8F, the area under the ROC curve (AUC) was 0.9156 (*p value* < 0.001), suggesting that KAT2A can effectively distinguish between NSCLC and normal tissue with high diagnostic accuracy. Furthermore, the correlation between METTL3 and KAT2A expression in NSCLC tissues was analyzed. The results demonstrated a positive association between the expression of KAT2A and METTL3 in NSCLC tissues (*r* = 0.5732, *p value* = 0.0255) (Fig. 8G). Meanwhile, qRT-PCR (Fig. 8H) and western blot (Fig. 8B) analysis showed that the expression of KAT2A in NSCLC cells was lowered by METTL3 silencing, whereas overexpression of METTL3 had the opposite results (Fig. 8I–B).

Based on the published MeRIP-seq data (GSE198288) of LUAD samples and tumor-adjacent normal samples, we observed the highest number of differential m6A peaks between LUAD samples and tumor-adjacent normal samples in the stop codon and 3’UTR (Fig. 8J), and the m6A occupancy of KAT2A was higher in LUAD tissues (Fig. 8K). Moreover, we re-analyzed the MeRIP-seq data (GSE55572) from METTL3-depleted A549 cells. The results show a decreased m6A occupancy of KAT2A from METTL3-depleted A549 cells compared to control (Fig. 8K), and the largest number of differential m6A peaks between METTL3-depleted A549 cells and control were found in the stop codon and 3’UTR (Fig. 8L). To explore this relationship further, we assessed the m6A levels of total RNAs in A549 and H460 cells. METTL3 knockdown resulted in a downregulation of global m6A modification level in both A549 and H460 cells compared to the control (Fig. 8M), while METTL3 overexpression increased global m6A modification level (Fig. 8N). Additionally, we used the SRAMP online tool [29] to predict potential m6A sites in KAT2A. The analysis revealed two significant m6A sites in the 3’UTR of KAT2A mRNA (Fig. 8O). To validate these predictions, we conducted MeRIP-qPCR and dual-luciferase reporter assays. MeRIP-qPCR showed that METTL3 knockdown significantly reduced m6A levels of fragments associated with the predicted site (Fig. 8O), and the dual-luciferase reporter assays confirmed that METTL3 knockdown decreased the activity of luciferase with wild-type KAT2A, but not with mutated KAT2A (Fig. 8P). These results imply that METTL3 regulated KAT2A expression by methylating the m6A site in 3’UTR of KAT2A mRNA in NSCLC cells.

### METTL3-mediated m6A mRNA modification of KAT2A mRNA promotes NSCLC progression

**Fig. 9:**
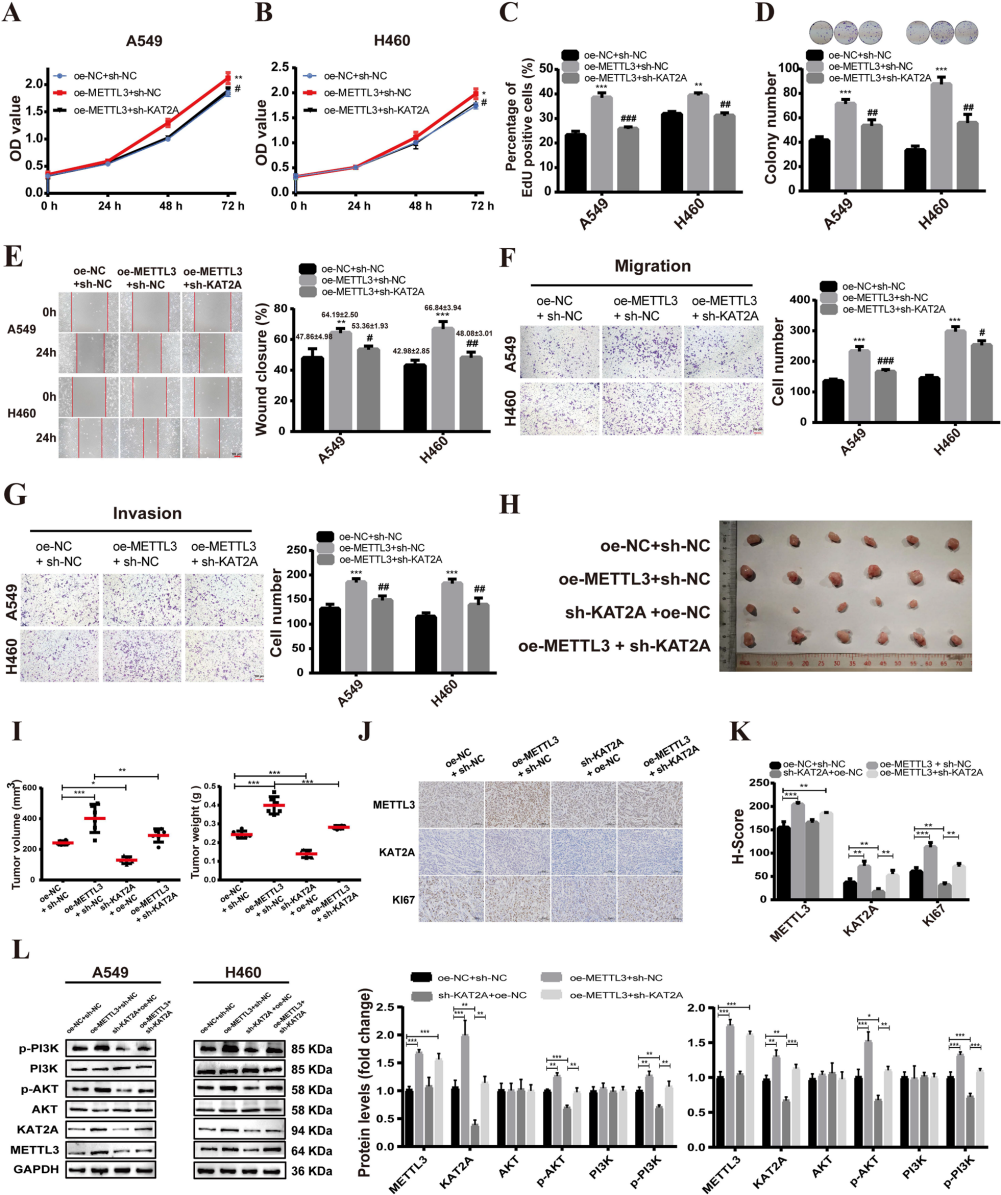
METTL3-mediated m6A mRNA modification of KAT2A mRNA promotes NSCLC progression. **A-C** CCK8 assays for A549 (**A**) and H460 (**B**) cells transfected with overexpression plasmid METTL3 alone or both oe-METTL3 and sh-NFIC. **D** Cell colony formation assays. **E** Scratch assay experiments on A549 and H460 cells. **F-G** Transwell migration (**F**) and invasion (**G**) assays. **H-I** The tumor volume and weight of tumors xenografted in nude mice. **J-K** IHC staining results and IHC score (H-score). **L** Western blot results. Bar = mean ± SD. *P < 0.05, **P < 0.01, ***P < 0.001, compared to oe-NC + sh-NC group; ^#^*P* < 0.05, ^##^*P* < 0.01, ^###^*P* < 0.001, compared to overexpression METTL3 (oe-METTL3 + sh-NC) group

Considering the METTL3 positively regulates KAT2A mRNA via m6A modification. Thus, we further investigated whether METTL3 promotes the progression of NSCLC via mediating KAT2A mRNA m6A modification. First, we investigated the effects of KAT2A on the progression of NSCLC. A549 and H460 cells were transfected with si-NC, si-KAT2A, sh-NC, or sh-KAT2A. qRT-PCR and western blot analyses revealed that transfection with siRNA-KAT2A or shRNA-KAT2A significantly decreased KAT2A expression compared to controls (Additional file 3: Fig. S1A-S1C). Cell proliferation, colony formation assays, cell migration, and invasion assays, revealed that KAT2A knockdown inhibited the malignant phenotype of A549 and H460 cells in vitro (Additional file 3: Fig. S1D-S1K). Next, rescue experiments show that KAT2A knockdown largely suppressed the promoting effect of overexpression of METTL3 on the malignant phenotype of A549 and H460 cells (Fig. 9A–G). In addition, in vivo experiments demonstrated that the overexpression of METTL3 significantly increased tumor size and weight compared to control groups, but this effect was inhibited by KAT2A knockdown (Fig. 9H–I). IHC staining revealed that overexpression of METTL3 increased the expression of KAT2A and ki67, which was counteracted by knockdown of KAT2A (Fig. 9J–K). Additionally, western blot analysis demonstrated that the METTL3 overexpression enhanced the phosphorylation of PI3K and AKT, which was recovered by KAT2A knockdown (Fig. 9L), suggesting that KAT2A knockdown impairs the activation of PI3K/AKT signaling pathway induced by overexpression of METTL3.

### NFIC negatively regulates the expression of KAT2A by directly binding to the KAT2A promotor region

**Fig. 10:**
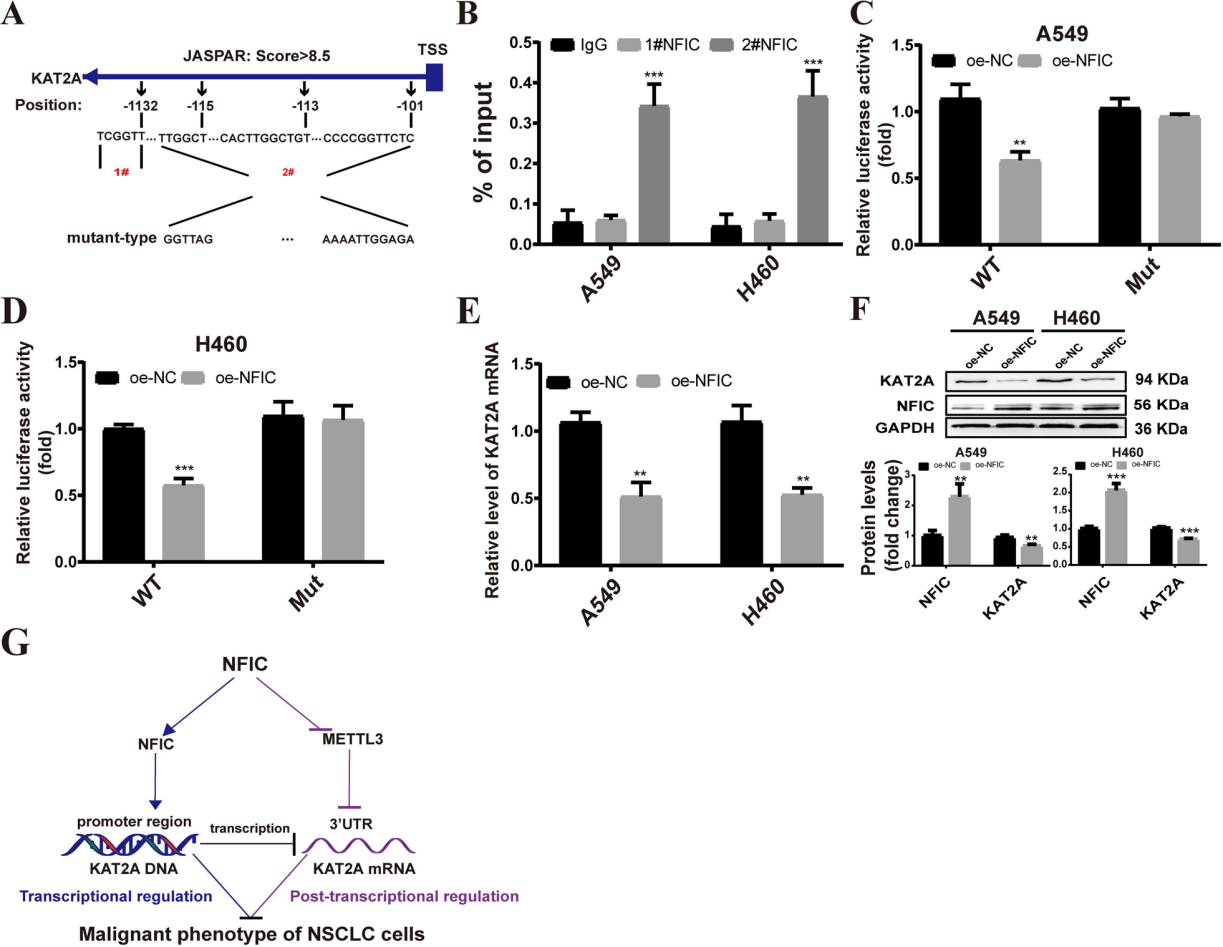
NFIC negatively regulates the expression of KAT2A by directly binding to KAT2A promotor region. **A** Prediction results of the binding of NFIC at the site of upstream the TSS of KAT2A. **B** ChIP-qPCR detected NFIC binding to KAT2A promoter region. **C-D** Assessment of KAT2A promoter activity after NFIC overexpression in A549 (**C**) and H460 (**D**) cells via dual‐luciferase reporter assay. **E–F** qRT‒PCR (**E**) and western blot (**F**) measurement of KAT2A expression in A549 and H460 cells.** G** An illustration of the molecular and functional mechanisms of NFIC in NSCLC cells. Bar = mean ± SD. **P < 0.01, ***P < 0.001

Based on the above data, NFIC modulates NSCLC progression by indirect regulation of KAT2A mRNA through METTL3-mediated m6A modification. Interestingly, significant differences in the chromatin features of KAT2A were identified between NSCLC and normal tissues (Fig. 1A). Therefore, we investigated whether NFIC binds to the KAT2A promoter region and directly regulates its transcription. The JASPAR online tool identified four potential NFIC binding sites at positions 101–1137 upstream of the KAT2A transcription start site (Fig. 10A). In order to validate these predicted binding sites, specific primers were designed to amplify the four sites, which were combined into a region of less than 200 base pairs, and ChIP-qPCR was performed to confirm the results. The ChIP-qPCR results showed a relative enrichment of NFIC at the METTL3 promoter (2#) (Fig. 10B). In order to further confirm the binding sites, the potential NFIC binding sites in the KAT2A promoter were mutated as shown in Fig. 10A. Dual-luciferase reporter assays demonstrated that overexpression of NFIC led to a decrease in luciferase activity with the wild-type KAT2A, but not with the mutated KAT2A, in A549 (Fig. 10C) and H460 cells (Fig. 10D). Additionally, the expression of KAT2A in the NFIC overexpression group were significantly decreased compared to the control group in A549 and H460 cells (Fig. 10E-F). These results suggested that the NFIC directly regulated KAT2A expression via binding to the promoter region of KAT2A in NSCLC cells.

## Discussion

Lung cancer is the most common malignancy worldwide, with non-small cell lung cancer (NSCLC) accounting for about 85% of all lung cancers [2, 30]. Despite advancements in diagnostic and therapeutic strategies, the prognosis for NSCLC remains poor [31]. Recently, there has been increasing research attention on the potential impact of genetic and epigenetic regulatory mechanisms at the levels of transcription and post-transcription on the development of human diseases [3–5]. Thus, further studies exploring the synergistic relationship between genetic and epigenetic regulatory mechanisms will yield valuable insights for preventing the development of NSCLC. In this study, transcription factor NFIC was screened using the chromatin property data (ATAC-seq, DNase-seq) and gene expression data (RNA-seq) from public databases, comparing NSCLC tissues with normal lung tissues. NFIC is a member of the NFI gene family, which comprises four genes (*NFIA*, *NFIB*, *NFIC*, and *NFIX*) [32]. Previous research has focused on investigating NFIC as a crucial regulator of tooth development, specifically its involvement in odontogenic cell proliferation, differentiation, and survival during root formation [33, 34]. In recent years, increasing number of studies have shown that NFIC plays an important role in various types of cancer, such as esophageal squamous cell cancer cells [35] breast cancer [36], gastric cancer [37], and bladder cancer [38]. However, research on NFIC in NSCLC mainly focuses on the bioinformatics analysis. Bioinformatics analysis conducted by Li et al. revealed an downregulation of NFIC expression in NSCLC [39, 40], and NFIC might be the key transcription factor in the development of lung squamous cell carcinoma [41]. Here, we observed significantly lower expression of NFIC in NSCLC tissues and the NSCLC cell lines, and overexpression of NFIC suppresses the malignant traits of NSCLC cells through inactivation of the PI3K/AKT pathway, suggesting that NFIC could serve as a potential biomarker and therapeutic target for NSCLC.

N6-methyladenosine (m6A) is an abundant post-transcriptional reversible modification that has a profound impact on the initiation and progression of cancer [10, 11]. As a key member of the m6A methyltransferase complex, METTL3 plays critical roles in regulating gene expression and affecting the progression of lung cancer [11, 42]. Lin et al. reported that METTL3 is significantly upregulated in primary human LUAD compared to adjacent normal tissues, and it promotes the growth, survival, and invasion of lung cancer cell lines [11]. Dou et al. also demonstrated that METTL3 promotes cell proliferation and colony formation in NSCLC [27]. In line with these findings, we found that METTL3 was significantly associated with prognosis in NSCLC patients, and knockdown of METTL3 suppresses NSCLC progression. Furthermore, we found that a significant negative correlation was observed between *METTL3* and the *NFIC* gene, and NFIC could negatively regulated the expression of METTL3 at the transcriptional level by binding to the promoter regions of METTL3. In addition, our investigation revealed that upregulation of NFIC led to a delay in the progression of NSCLC by effectively downregulating the expression of METTL3. Previous studies have primarily focused on investigating the regulatory relationship between METTL3 and NFIC in relation to tooth root formation [43]. Sheng et al. found that METTL3-mediated m6A mRNA methylation modulates tooth root formation by affecting NFIC translation [43]. Therefore, our findings significantly contribute to a deeper understanding of the intricate relationship between transcription factors and m6A in the context of cancer development.

The role of METTL3 in regulating cancer progression through m6A modification of target genes has been extensively studied [28]. In this study, we screened KAT2A, a histone acetyltransferase, as a target gene of METTL3 by using the WGCNA and integrating multi-omics data. It has been shown that the significance of KAT2A in various cancers, including liver cancer [44], breast cancer [45], and colon Cancer [46]. As for the regulatory mechanism, previous studies mainly focused on the downstream regulatory mechanism of KAT2A [47, 48]. For instance, Chen et al. found that elevated expression of KAT2A promotes the growth of NSCLC by upregulating E2F1, Cyclin D1, and Cyclin E1 [49]. However, the m6A modifications governing KAT2A function in cancers and how KAT2A expression may be modulated by m6A modification in cancer remain largely unclear. Here, METTL3 knockdown reduced the expression of KAT2A by methylating the m6A site in the 3’UTR of KAT2A mRNA in NSCLC cells. Moreover, METTL3-mediated m6A modification of KAT2A mRNA promotes progression of NSCLC. Interestingly, we also discovered that NFIC negatively regulates the expression of the KAT2A by directly binding to the KAT2A promoter region. Notably, inhibitors targeting the m6A regulator [50, 51] and transcription factor [52, 53] have shown potential as cancer therapies. Treatment with catalytic inhibitor of METTL3 (STM2457) leads to reduced acute myeloid leukemia growth, and an increase in differentiation and apoptosis [50]. In addition, a small number of transcription factors, such as the estrogen and androgen receptors that drive breast and prostate cancer, respectively, have been drugged by small molecules [53]. Therefore, our study on the synergistic effects of transcriptional regulation through the action of transcription factors and post-transcriptional regulation via m6A will provide new insights into the mechanism of precision treatment for NSCLC (Fig. 10G).

There is a limitation to this study. Based on multi-omics data, we identified the transcription factor NFIC and its potential target genes. However, we primarily focused on m6A-related genes and did not analyze other genes, which may have caused bias in gene selection.

## Conclusions

In this study, we demonstrated that NFIC is essential for NSCLC progression by connecting gene transcription with m6A modification. The regulatory role of NFIC, which suppresses the malignant phenotype of NSCLC cells at both the transcriptional and post-transcriptional levels, offers significant insights into the interplay between transcription factors and m6A modification in cancer. A deeper understanding of the genetic and epigenetic regulatory mechanisms of tumorigenesis will help to a foundation for development of personalized treatment strategies.

### Supplementary Information

Additional file 1: Table S1. The sequences of siRNAs and shRNAs. **Table S2.** Primer sequences of target genes. Table S3. The sequences of ChIP-qPCR and MeRIP-qPCR primers.

Additional file 2: Western blot raw data.

Additional file 3: Fig. S1 **A-C**. qRT-PCR and western blot analysis were performed to show KAT2A knockdown. **D-E** Proliferation of A549 (**D**) and H460 **(E**) cells following KAT2A knockdown was determined using CCK8 assays. **F-G** Colony formation assay was performed in A549 (**F**) and H460 (**G**) cells after knockdown of KAT2A. **H-K** Cell migratory** (H-I)** and invasive **(J-K)** abilities were detected using transwell assays in A549 and H460 cells. Bar = mean ± SD. **P < 0.01, ***P < 0.001, compared to si-NC group; ^###^*P* < 0.001, compared to sh-NC group.

## Data Availability

The datasets used and/or analyzed during the current study are available from the corresponding author on reasonable request.

## Abbreviations

NFIC: Nuclear factor I C
METTL3: Methyltransferase 3
NSCLC: Non-small cell lung cancer
KAT2A: Lysine acetyltransferase 2A
m6A: N6-methyladenosine
MeRIP-seq: Methylated RNA immunoprecipitation sequencing
WGCNA: Weighted correlation network analysis
GO: Gene Ontology
KEGG: Kyoto Encyclopedia of Genes and Genomes
TF: Transcription factor
qRT‒PCR: Quantitative real-time polymerase chain reaction
CDS: Coding sequences
TSS: Transcription start site

## References

[1] Smith D, Raices M, Cayol F, Corvatta F, Caram L, Dietrich A (2022). Is the neutrophil-to-lymphocyte ratio a prognostic factor in non-small cell lung cancer patients who receive adjuvant chemotherapy?. Semin Oncol.

[2] Bray F, Ferlay J, Soerjomataram I, Siegel RL, Torre LA, Jemal A (2018). Global cancer statistics 2018: GLOBOCAN estimates of incidence and mortality worldwide for 36 cancers in 185 countries. CA A Cancer J Clinicians.

[3] Suzuki M, Jing Q, Lia D, Pascual M, McLellan A, Greally JM (2010). Optimized design and data analysis of tag-based cytosine methylation assays. Genome Biol.

[4] Song T, Yang Y, Jiang S, Peng J (2020). Novel insights into adipogenesis from the perspective of transcriptional and RNA n6-methyladenosine-mediated post-transcriptional regulation. Adv Sci.

[5] Farooqi AA, Fayyaz S, Poltronieri P, Calin G, Mallardo M (2022). Epigenetic deregulation in cancer: enzyme players and non-coding RNAs. Semin Cancer Biol.

[6] Chen JF, Yan Q (2021). The roles of epigenetics in cancer progression and metastasis. Biochem J.

[7] Dawson MA (2017). The cancer epigenome: concepts, challenges, and therapeutic opportunities. Science.

[8] Topper MJ, Vaz M, Chiappinelli KB, DeStefano Shields CE, Niknafs N, Yen RC (2017). Epigenetic therapy ties MYC depletion to reversing immune evasion and treating lung cancer. Cell.

[9] Zhao Z, Meng J, Su R, Zhang J, Chen J, Ma X (2020). Epitranscriptomics in liver disease: basic concepts and therapeutic potential. J Hepatol.

[10] Hu C, Liu J, Li Y, Jiang W, Ji D, Liu W (2022). Multifaceted roles of the N(6)-Methyladenosine RNA methyltransferase METTL3 in cancer and immune microenvironment. Biomolecules.

[11] Lin S, Choe J, Du P, Triboulet R, Gregory RI (2016). The m(6)A methyltransferase METTL3 promotes translation in human cancer cells. Mol Cell.

[12] Kuppers DA, Arora S, Lim Y, Lim AR, Carter LM, Corrin PD (2019). N(6)-methyladenosine mRNA marking promotes selective translation of regulons required for human erythropoiesis. Nat Commun.

[13] Liu XM, Mao Y, Wang S, Zhou J, Qian SB (2022). METTL3 modulates chromatin and transcription dynamics during cell fate transition. Cell Mol Life Sci CMLS.

[14] Deng S, Zhang J, Su J, Zuo Z, Zeng L, Liu K (2022). RNA m(6)A regulates transcription via DNA demethylation and chromatin accessibility. Nat Genet.

[15] Shi K, Sa R, Dou L, Wu Y, Dong Z, Fu X (2023). METTL3 exerts synergistic effects on m6A methylation and histone modification to regulate the function of VGF in lung adenocarcinoma. Clin Epigenet.

[16] Stein GS, Stein JL, Van Wijnen AJ, Lian JB, Montecino M, Croce CM (2010). Transcription factor-mediated epigenetic regulation of cell growth and phenotype for biological control and cancer. Adv Enzyme Regul.

[17] Bakshi R, Zaidi SK, Pande S, Hassan MQ, Young DW, Montecino M (2008). The leukemogenic t(8;21) fusion protein AML1-ETO controls rRNA genes and associates with nucleolar-organizing regions at mitotic chromosomes. J Cell Sci.

[18] Wang Z, Tu K, Xia L, Luo K, Luo W, Tang J (2019). The open chromatin landscape of non-small cell lung carcinoma. Can Res.

[19] Shi K, Wang B, Dou L, Wang S, Fu X, Yu H (2022). Integrated bioinformatics analysis of the transcription factor-mediated gene regulatory networks in the formation of spermatogonial stem cells. Front Physiol.

[20] Heinz S, Benner C, Spann N, Bertolino E, Lin YC, Laslo P (2010). Simple combinations of lineage-determining transcription factors prime cis-regulatory elements required for macrophage and B cell identities. Mol Cell.

[21] Sherman BT, Hao M, Qiu J, Jiao X, Baseler MW, Lane HC (2022). DAVID: a web server for functional enrichment analysis and functional annotation of gene lists (2021 update). Nucleic Acids Res.

[22] Tsompana M, Buck MJ (2014). Chromatin accessibility: a window into the genome. Epigenet Chromatin.

[23] Frank CL, Liu F, Wijayatunge R, Song L, Biegler MT, Yang MG (2015). Regulation of chromatin accessibility and Zic binding at enhancers in the developing cerebellum. Nat Neurosci.

[24] Grandi FC, Modi H, Kampman L, Corces MR (2022). Chromatin accessibility profiling by ATAC-seq. Nat Protoc.

[25] Liu Y, Fu L, Kaufmann K, Chen D, Chen M (2019). A practical guide for DNase-seq data analysis: from data management to common applications. Brief Bioinform.

[26] Xu L, Zhou L, Yan C, Li L (2022). Emerging role of N6-methyladenosine RNA methylation in lung diseases. Exp Biol Med (Maywood).

[27] Dou X, Wang Z, Lu W, Miao L, Zhao Y (2022). METTL3 promotes non-small cell lung cancer (NSCLC) cell proliferation and colony formation in a m6A-YTHDF1 dependent way. BMC Pulm Med.

[28] Liu X, He H, Zhang F, Hu X, Bi F, Li K (2022). m6A methylated EphA2 and VEGFA through IGF2BP2/3 regulation promotes vasculogenic mimicry in colorectal cancer via PI3K/AKT and ERK1/2 signaling. Cell Death Dis.

[29] Zhou Y, Zeng P, Li YH, Zhang Z, Cui Q (2016). SRAMP: prediction of mammalian N6-methyladenosine (m6A) sites based on sequence-derived features. Nucleic Acids Res.

[30] Bajbouj K, Al-Ali A, Ramakrishnan RK, Saber-Ayad M, Hamid Q (2021). Histone modification in NSCLC: molecular mechanisms and therapeutic targets. Int J Mol Sci.

[31] Travis WD, Brambilla E, Noguchi M, Nicholson AG, Geisinger KR, Yatabe Y (2011). International association for the study of lung cancer/american thoracic society/european respiratory society international multidisciplinary classification of lung adenocarcinoma. Journal Thoracic Oncol Official Publ Int Assoc Study Lung Cancer.

[32] Zhang F, Liang M, Zhao C, Fu Y, Yu S (2019). NFIC promotes the vitality and osteogenic differentiation of rat dental follicle cells. J Mol Histol.

[33] Lee DS, Park JT, Kim HM, Ko JS, Son HH, Gronostajski RM (2009). Nuclear factor I-C is essential for odontogenic cell proliferation and odontoblast differentiation during tooth root development. J Biol Chem.

[34] Oh HJ, Lee HK, Park SJ, Cho YS, Bae HS, Cho MI (2012). Zinc balance is critical for NFI-C mediated regulation of odontoblast differentiation. J Cell Biochem.

[35] Wang H, Shi X, Wu S (2020). miR-550a-3/NFIC plays a driving role in esophageal squamous cell cancer cells proliferation and metastasis partly through EMT process. Mol Cell Biochem.

[36] Lee HK, Lee DS, Park JC (2015). Nuclear factor I-C regulates E-cadherin via control of KLF4 in breast cancer. BMC Cancer.

[37] Xu G, Zhang Y, Li N, Wu Y, Zhang J, Xu R (2020). LBX2-AS1 up-regulated by NFIC boosts cell proliferation, migration and invasion in gastric cancer through targeting miR-491-5p/ZNF703. Cancer Cell Int.

[38] Liang X, Gao J, Wang Q, Hou S, Wu C (2020). ECRG4 represses cell proliferation and invasiveness via NFIC/OGN/NF-κB signaling pathway in bladder cancer. Front Genet.

[39] Bhattacharjee A, Richards WG, Staunton J, Li C, Monti S, Vasa P (2001). Classification of human lung carcinomas by mRNA expression profiling reveals distinct adenocarcinoma subclasses. Proc Natl Acad Sci USA.

[40] Li Y, Sun C, Tan Y, Li L, Zhang H, Liang Y (2020). Transcription levels and prognostic significance of the NFI family members in human cancers. PeerJ.

[41] Zhang F, Chen X, Wei K, Liu D, Xu X, Zhang X (2017). Identification of key transcription factors associated with lung squamous cell carcinoma. Med Sci Monit.

[42] Zeng C, Huang W, Li Y, Weng H (2020). Roles of METTL3 in cancer: mechanisms and therapeutic targeting. J Hematol Oncol.

[43] Sheng R, Wang Y, Wu Y, Wang J, Zhang S, Li Q (2021). METTL3-mediated m(6) A mRNA methylation modulates tooth root formation by affecting NFIC translation. J Bone Mineral Res Offic J Am Soc Bone Mineral Res.

[44] Majaz S, Tong Z, Peng K, Wang W, Ren W, Li M (2016). Histone acetyl transferase GCN5 promotes human hepatocellular carcinoma progression by enhancing AIB1 expression. Cell Biosci.

[45] Zhao L, Pang A, Li Y (2018). Function of GCN5 in the TGF-β1-induced epithelial-to-mesenchymal transition in breast cancer. Oncol Lett.

[46] Yin YW, Jin HJ, Zhao W, Gao B, Fang J, Wei J (2015). The histone acetyltransferase GCN5 expression is elevated and regulated by c-Myc and E2F1 transcription factors in human colon cancer. Gene Expr.

[47] Li T, Su L, Lei Y, Liu X, Zhang Y, Liu X (2015). DDIT3 and KAT2A proteins regulate TNFRSF10A and TNFRSF10B expression in endoplasmic reticulum stress-mediated apoptosis in human lung cancer cells. J Biol Chem.

[48] Han X, Chen J (2022). KAT2A affects tumor metabolic reprogramming in colon cancer progression through epigenetic activation of E2F1. Hum Cell.

[49] Chen L, Wei T, Si X, Wang Q, Li Y, Leng Y (2013). Lysine acetyltransferase GCN5 potentiates the growth of non-small cell lung cancer via promotion of E2F1, cyclin D1, and cyclin E1 expression. J Biol Chem.

[50] Yankova E, Blackaby W, Albertella M, Rak J, De Braekeleer E, Tsagkogeorga G (2021). Small-molecule inhibition of METTL3 as a strategy against myeloid leukaemia. Nature.

[51] Jin N, George TL, Otterson GA, Verschraegen C, Wen H, Carbone D (2021). Advances in epigenetic therapeutics with focus on solid tumors. Clin Epigenetics.

[52] Samarasinghe KTG, Jaime-Figueroa S, Burgess M, Nalawansha DA, Dai K, Hu Z (2021). Targeted degradation of transcription factors by TRAFTACs: TRAnscription factor TArgeting chimeras. Cell Chem Biol.

[53] Rice MA, Malhotra SV, Stoyanova T (2019). Second-generation antiandrogens: from discovery to standard of care in castration resistant prostate cancer. Front Oncol.

