# Supplementary material for: NFIC mediates m6A mRNA methylation to orchestrate transcriptional and post-transcriptional regulation to represses malignant phenotype of non-small cell lung cancer cells
Source: Cancer Cell Int. 2024 Jun 28;24:223. doi: 10.1186/s12935-024-03414-1 (PMC11212411; doi:10.1186/s12935-024-03414-1)
Supplement: Supplementary file 1 — Additional file 1: Table S1. The sequences of siRNAs and shRNAs. Table S2. Primer sequences of target genes. Table S3. The sequences of ChIP-qPCR and MeRIP-qPCR primers. [file 12935_2024_3414_MOESM1_ESM.docx]

**Table S1. The sequences of siRNAs and shRNAs**

| Target | Sequence (5’-3’) |
| --- | --- |
| siRNA - control | UUCUCCGAACGUGUCACG UTT |
| siRNA - METTL3-1 | CCUGCAAGUAUGUUCACUATT |
| siRNA - METTL3-2 | GCUGCACUUCAGACGAAUUTT |
| siRNA - KAT2A-1 | GCUCUACACAACCCUCAAATT |
| siRNA- KAT2A-2 | GGAAAUGCAUCCUGCAGAUTT |
| siRNA - KAT2A-3 | GAAGCUGAUUGAGCGCAAATT |
| shRNA - control | TTCTCCGAACGTGTCACGT |
| shRNA - METTL3 | GCTGCACTTCAGACGAATTAT |
| shRNA - KAT2A | GCTGAACTTTGTGCAGTACAA |

**Table S2. Primer sequences of target genes**

| Target | Sequence (5’-3’) |
| --- | --- |
| GAPDH | F:5’-GCACCGTCAAGGCTGAGAAC-3’ |
|  | R:5’-TGGTGAAGACGCCAGTGGA-3’ |
| NFIC | F:5'-TGGCGGCGATTACTACACTTCG-3' |
|  | R:5’-GGCTGTTGAATGGTGACTTGTCC -3’ |
| METTL3 | F:5’-CAAGCTGCACTTCAGACGAA-3’ |
|  | R:5’-GCTTGGCGTGTGGTCTTT-3’ |
| FTO | F:5’-GGTCGAGTTTGAGTGGCTGA-3’ |
|  | R:5’-GTTCGGGCAATTCGTGACTG-3’ |
| HNRNPC | F: 5’-CAGAACACCCTTCTCCGTCC-3’ |
|  | R: 5’-ACGTTTCGAGGGCACTACAG-3’ |
| HNRNPA2B1 | F: 5’-GGAGTGGAAGAGGAGGCAAC-3’ |
|  | R: 5’-CAGGTCCTCCTCCATACCCA-3’ |
| KAT2A | F:5’-AAGCTAGGGGTCTTCTCGGC-3’ |
|  | R:5’-TTCAGCACACCCTGCTCAAT-3’ |
| ATAT1 | F:5’-ATCCCCCAATACAGGCAACC-3’ |
|  | R:5’-ACCTGGCGTTGAGTATGTCC-3’ |
| MSH5 | F:5’-CTACTCAAGGCCGCGTTACT-3’ |
|  | R:5’-CTGCTTTCGCCACCTGGTT-3’ |
| ATAD3B | F:5’-TGAACCTGCTGCACACACTA-3’ |
|  | R:5’-CTTCCTCCTCTCCTCAGCCT-3’ |
| POLG2 | F:5’-GGCTACCTTATGGCCTTGCT-3’ |
|  | R:5’-GCCCTTGACAAACCTGTCTT-3’ |

**Table S3. The sequences of ChIP-qPCR and MeRIP-qPCR primers**

| Target | Sequence (5’-3’) |
| --- | --- |
| METTL3 (ChIP-qPCR) | F: 5'-GTGAAACCCCATCTCTATTA-3'  F: 5'-ACTGCAACCTCCACCTCCTG-3' |
| KAT2A-1 (ChIP-qPCR) | F: 5'-TCTCCGCCCTTCCCCACCTT-3'  R: 5'-CCGCCCTCCCCTGGCTCTGT-3' |
| KAT2A-2 (ChIP-qPCR) | F: 5'-CAGACCCCACGATTCTCCCC-3'  R: 5'-CCTTCCCCTCACTGCCTCCA-3' |
| KAT2A (MeRIP-qPCR) | F: 5'-TCCACCTCGGATTCTGATCT-3'  R: 5'-CACACAGTGAAGGCTGGGAC-3' |
