# Supplementary figures and images for: NFIC mediates m6A mRNA methylation to orchestrate transcriptional and post-transcriptional regulation to represses malignant phenotype of non-small cell lung cancer cells
Source: Cancer Cell Int. 2024 Jun 28;24:223. doi: 10.1186/s12935-024-03414-1 (PMC11212411; doi:10.1186/s12935-024-03414-1)

Fig. 2G

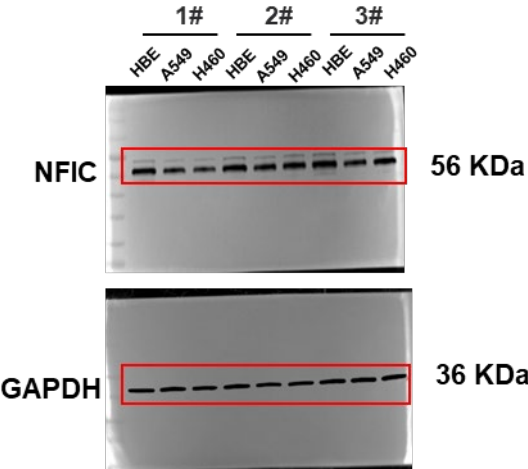

Fig. 3B

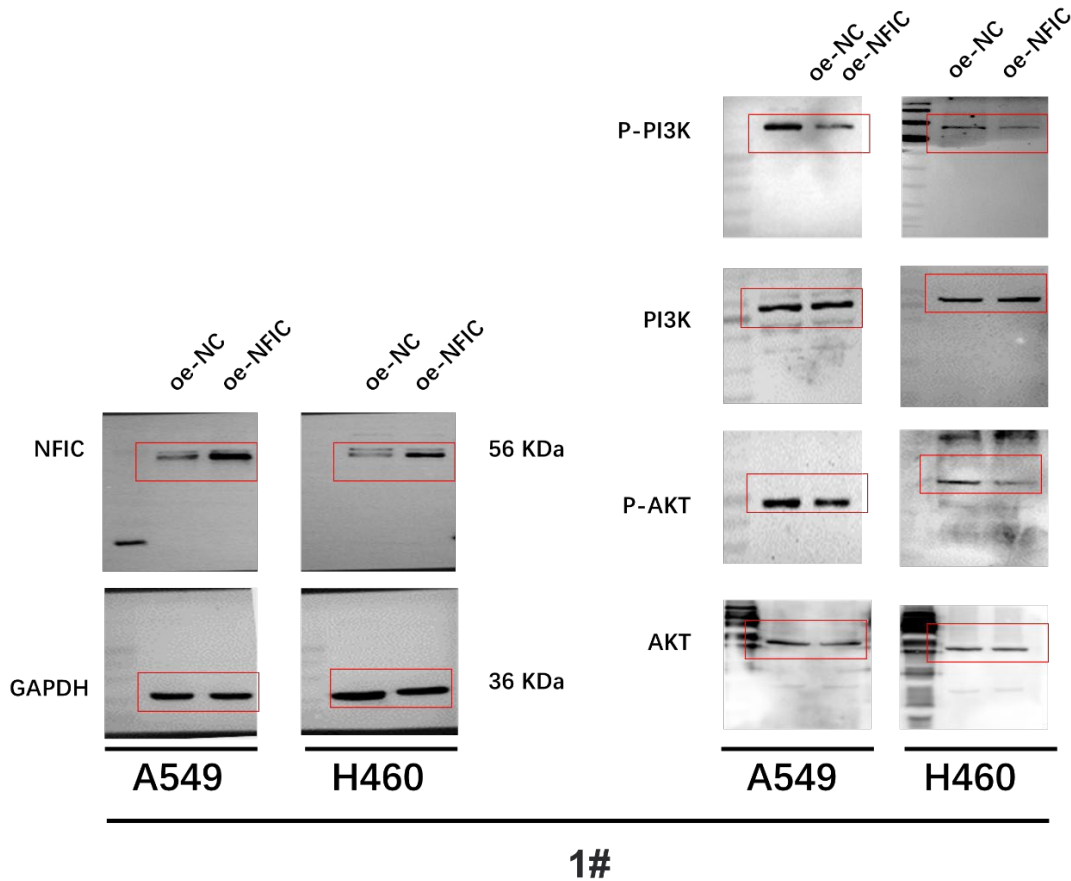

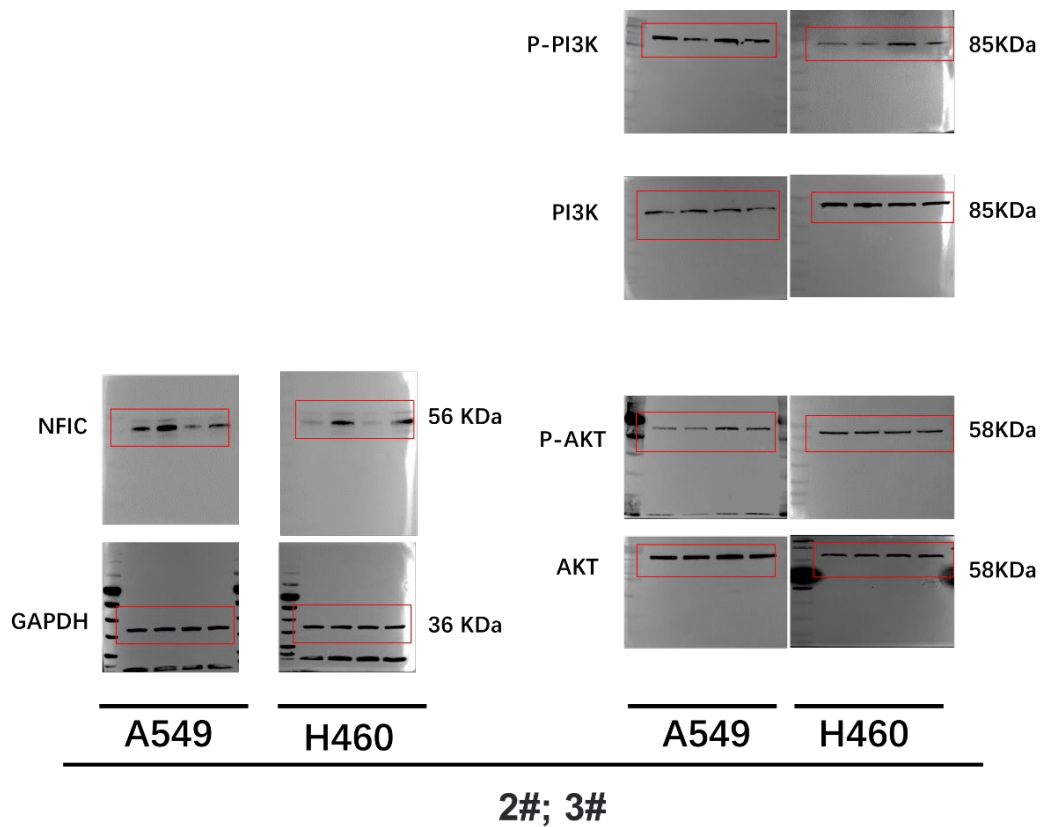

**Fig. 4I**

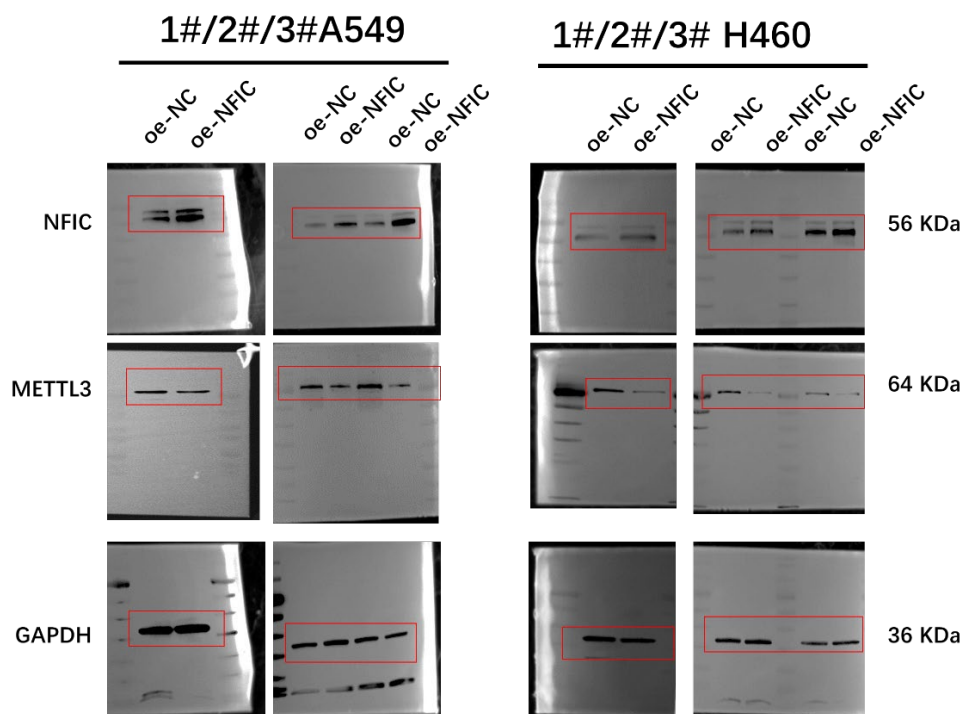

Fig. 5F

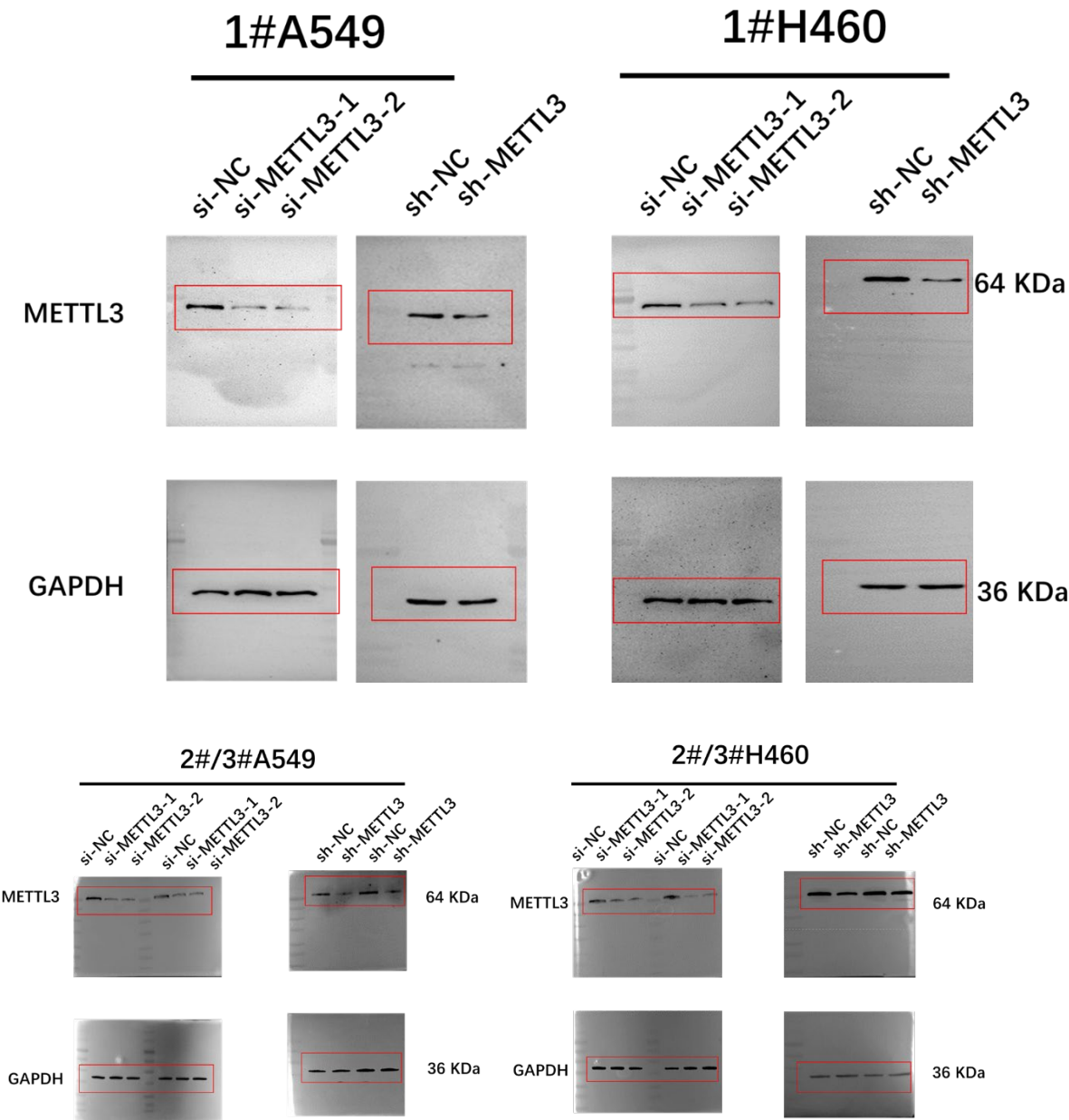

Fig. 8B

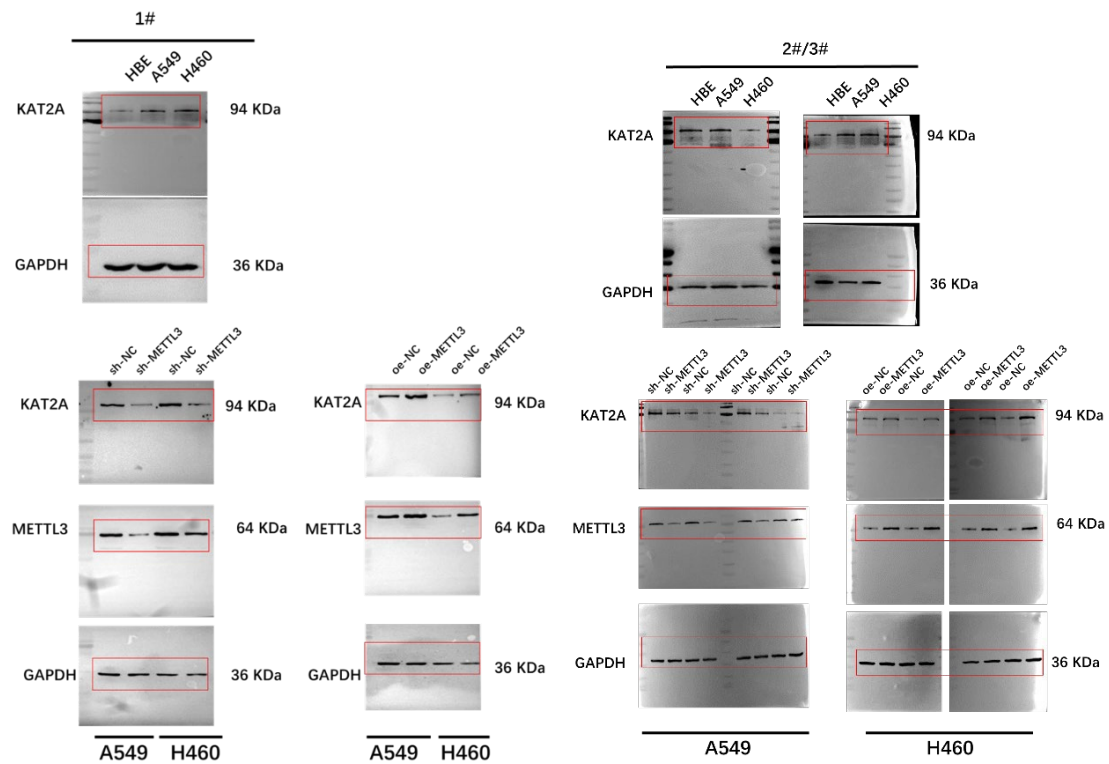

Fig. 9L

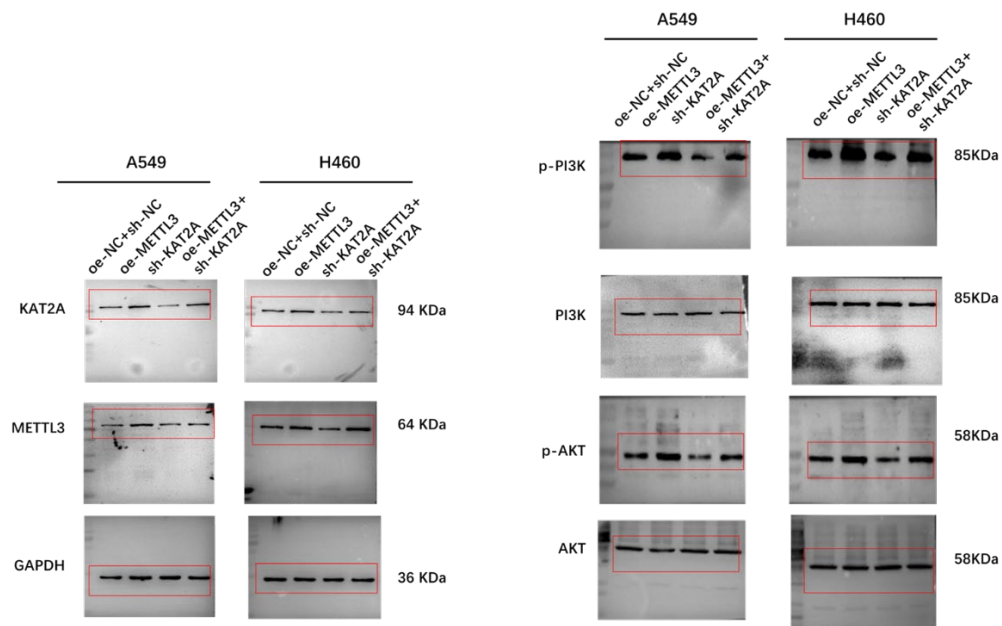

2#/3#

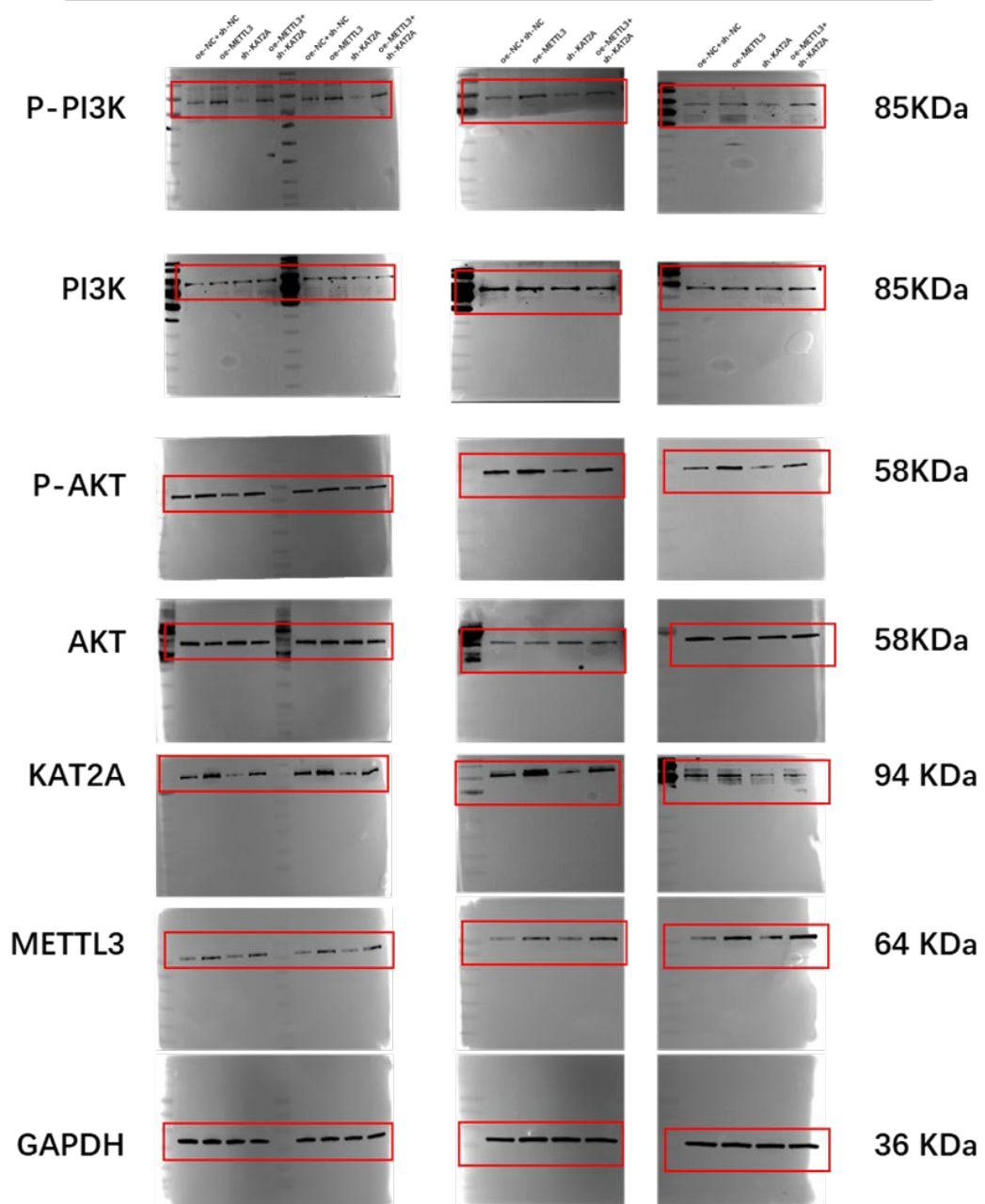

**Fig. 10F**

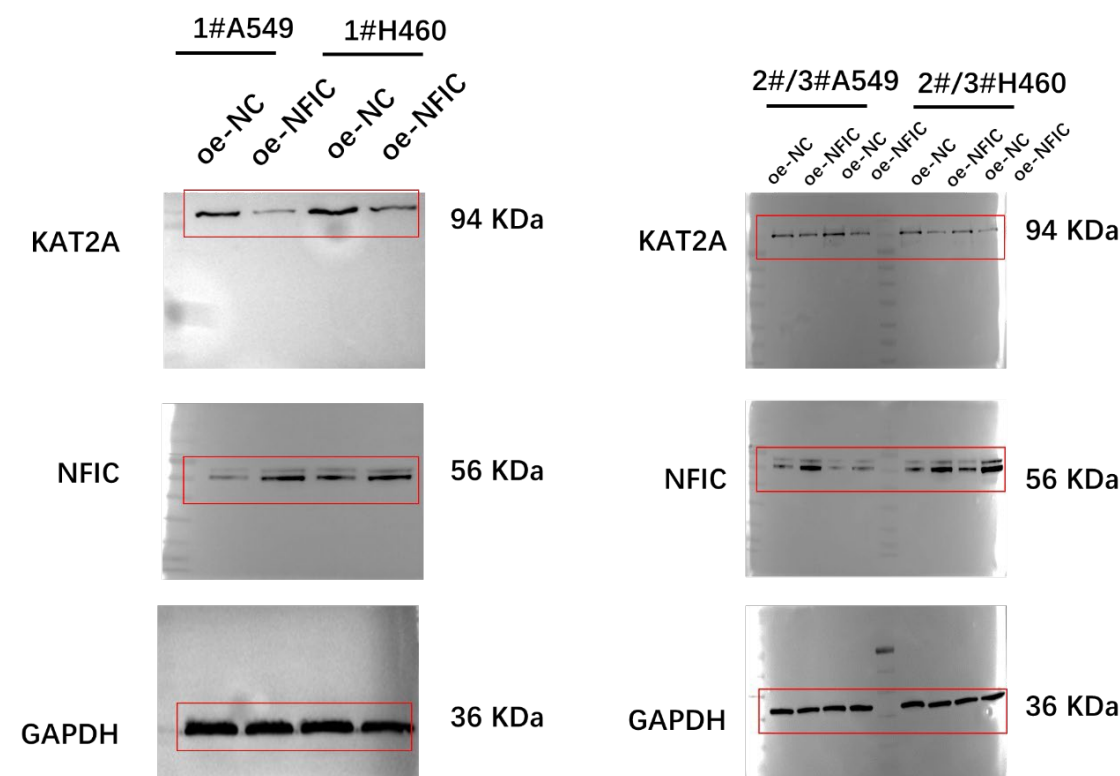

**Additional file 3 Fig. S1B**

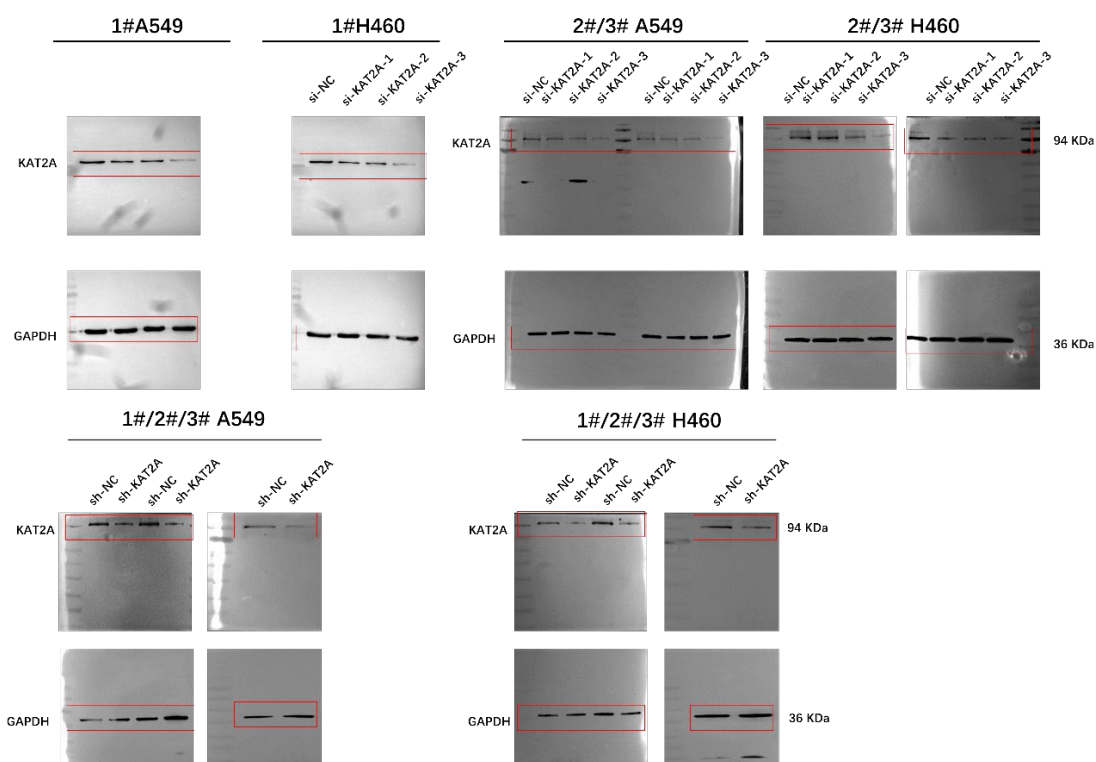

Supplement: Supplementary file 2 — Additional file 2: Western blot raw data. [file 12935_2024_3414_MOESM2_ESM.pdf]

**A**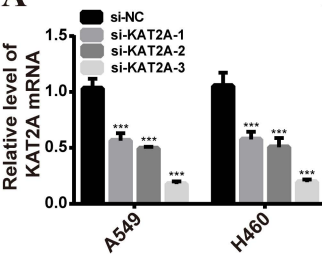**B**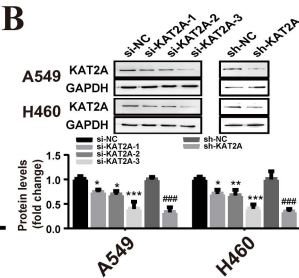**C**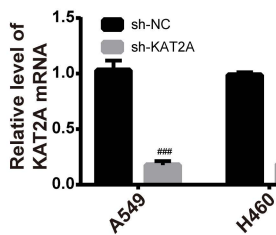**D**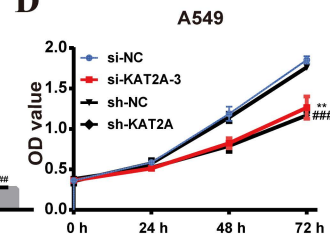**E**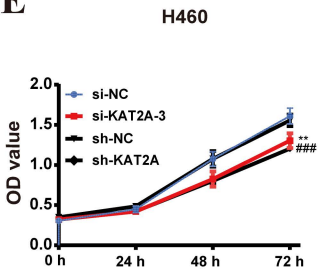**F**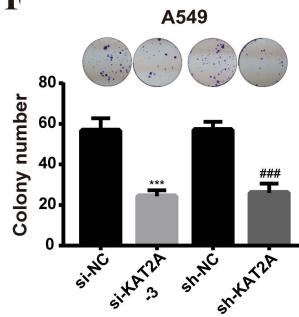**G**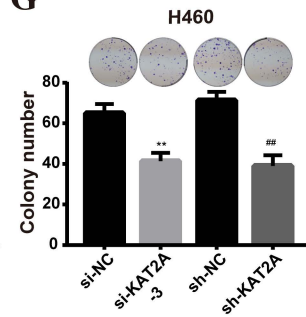**H**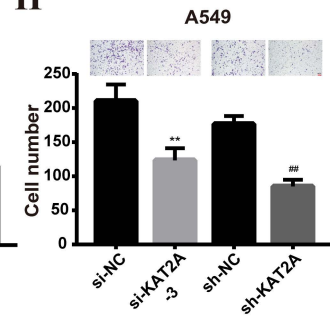**I**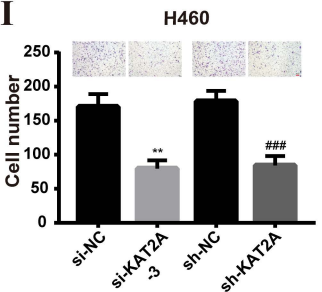**J**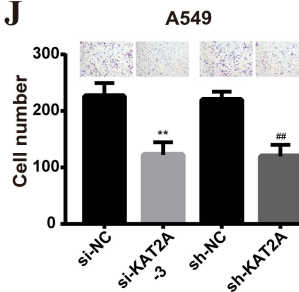**K**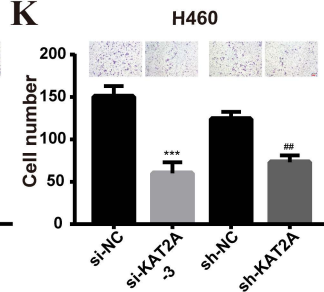

Supplement: Supplementary file 3 — Additional file 3: Fig. S1 A-C. qRT-PCR and western blot analysis were performed to show KAT2A knockdown. D-E Proliferation of A549 (D) and H460 (E) cells following KAT2A knockdown was determined using CCK8 assays. F-G Colony formation assay was performed in A549 (F) and H460 (G) cells after knockdown of KAT2A. H-K Cell migratory (H-I) and invasive (J-K) abilities were detected using transwell assays in A549 and H460 cells. Bar = mean ± SD. **P < 0.01, ***P < 0.001, compared to si-NC group; ###P < 0.001, compared to sh-NC group. [file 12935_2024_3414_MOESM3_ESM.pdf]
